# Regulation of Proline Accumulation and Protein Secretion in Sorghum under Combined Osmotic and Heat Stress

**DOI:** 10.3390/plants13131874

**Published:** 2024-07-06

**Authors:** Samkelisiwe P. Ngwenya, Sellwane J. Moloi, Nemera G. Shargie, Adrian P. Brown, Stephen Chivasa, Rudo Ngara

**Affiliations:** 1 Department of Plant Sciences, University of the Free State, Qwaqwa Campus, P. Bag X13, Phuthaditjhaba 9866, South Africa; samkeh120@gmail.com (S.P.N.); moloisj@ufs.ac.za (S.J.M.); 2 Agricultural Research Council-Grain Crops Institute, P. Bag X1251, Potchefstroom 2520, South Africa; shargien@arc.agric.za; 3 Department of Biosciences, Durham University, South Road, Durham DH1 3LE, UK; a.p.brown@durham.ac.uk (A.P.B.); stephen.chivasa@durham.ac.uk (S.C.)

**Keywords:** *Sorghum bicolor*, cell suspension cultures, secreted proteins, secretome, extracellular matrix, combined stress, proteomics, iTRAQ

## Abstract

Plants reprogramme their proteome to alter cellular metabolism for effective stress adaptation. Intracellular proteomic responses have been extensively studied, and the extracellular matrix stands as a key hub where peptide signals are generated/processed to trigger critical adaptive signal transduction cascades inaugurated at the cell surface. Therefore, it is important to study the plant extracellular proteome to understand its role in plant development and stress response. This study examined changes in the soluble extracellular sub-proteome of sorghum cell cultures exposed to a combination of sorbitol-induced osmotic stress and heat at 40 °C. The combined stress significantly reduced metabolic activity and altered protein secretion. While cells treated with osmotic stress alone had elevated proline content, the osmoprotectant in the combined treatment remained unchanged, confirming that sorghum cells exposed to combined stress utilise adaptive processes distinct from those invoked by the single stresses applied separately. Reactive oxygen species (ROS)-metabolising proteins and proteases dominated differentially expressed proteins identified in cells subjected to combined stress. ROS-generating peroxidases were suppressed, while ROS-degrading proteins were upregulated for protection from oxidative damage. Overall, our study provides protein candidates that could be used to develop crops better suited for an increasingly hot and dry climate.

## 1. Introduction

Plants encounter a wide range of abiotic stresses, which limit their growth, yield, and geographical distribution patterns [1,2]. These stress factors may occur individually, sequentially, or simultaneously, causing adverse effects on plant physiology and metabolism [3,4,5]. While stress combinations such as ozone and drought or high CO_2_ and drought may positively influence the growth and performance of plants, others, including drought and heat, often cause irreparable damage and devastating yield losses [3,4]. In addition, drought and heat are often inseparable in the field, particularly in the arid and semi-arid tropics, and their negative impact on crop productivity is aggravated by climate change and global warming events [6].

The climate in sub-Saharan Africa, especially Southern Africa, is increasingly becoming hotter and drier, and drought and heat stresses are among the leading causes of crop failure and food insecurity in the region [7,8]. Of course, the economic and social consequences of climate change might differ between regions [7,8]. Nevertheless, rural populations in Africa are particularly vulnerable to global warming scenarios due to their reliance on rainfed, low-technology-based farming practices for their livelihoods [9,10]. Therefore, understanding plant responses to combined heat and water deficit stress is a prerequisite for developing resilient crops suitable for a warmer and drier climate [11].

Morpho-physiological and molecular responses of plants to individual stresses of drought [1,12] and heat [2,13] have been extensively studied and reviewed, and some drought [14] and heat [15] resilient food crops have been developed. It is known that signalling pathways of different abiotic stresses may crosstalk and converge, resulting in common plant responses to diverse stresses [16,17,18]. Nonetheless, individual stresses of drought and heat also pose specific challenges to plants that require unique sets of plant responses in mitigation. For example, drought-stressed plants reduce transpiration-dependent water loss by limiting stomatal conductance [1], while heat-stressed plants benefit from evaporative cooling achieved through increased stomatal conductance [2]. Because of these potentially antagonistic effects of stressors on plants, several studies are now exploring ways in which plants adapt to stress combinations [3,4,5,19], including combined drought and heat stress [20,21,22]. Rivero et al. [23] described such responses as complex and possibly involving selective, additive, antagonistic, and unique regulatory mechanisms to combat the newly developed physiological and metabolic strains of stress combinations. This increased complexity in plant responses has been demonstrated in transcriptomics [24,25,26,27,28,29,30], proteomics [31,32,33,34,35] and metabolomics [21,25,36,37,38,39] studies of various plant species under combined drought and heat stress.

Results of the “omics” studies highlight an overlap of stress-responsive transcripts, proteins, and metabolites against drought, heat, and their combined stress. However, combined heat and drought stress also induces unique sets of stress-adaptive responses compared to the stresses applied separately [23]. Furthermore, combined drought and heat stress causes even greater detrimental effects on plant growth and reproductive capacity than each of the individual stresses [4,34,40]. Such observations highlight the need to study combined drought and heat stress effects on plants as a new form of stress that differs from its individual stress components [3]. To date, however, proteomic studies on combined drought and heat stress have mainly focused on the intracellular leaf proteome of *Carissa spinarum* [31], wild barley (*Hordeum spontaneum*) [33], maize (*Zea mays*) [32,34], and soybean (*Glycine max*) [35], with no equivalent investigations on secreted proteins.

Secreted proteins, also known as the secretome, are integral to cell structure and function [41,42]. These proteins are translocated from the cell constitutively or in response to biotic or abiotic stress factors [42,43,44]. Functionally, secreted proteins serve diverse roles in cell structure, signalling, intercellular communication, and defence processes [41,42,44]. As such, the plant secretome contains potentially valuable stress-responsive proteins that could serve as candidates for developing stress-resilient crops. Therefore, this study investigated the effects of combined osmotic and heat stress on secreted proteins and on osmolyte profiles of sorghum (*Sorghum bicolor*) cell suspension cultures to gain insights into how metabolism is altered under combined stress. We specifically sought to understand the similarities and differences between sorghum responses to heat and osmotic stressed applied individually and in combination. We chose to use sorghum due to its natural drought and heat tolerance compared to other cereals such as maize, rice (*Oryza sativa*), and wheat (*Triticum aestivum*) [45,46]. Furthermore, sorghum is considered a good model system for understanding drought and heat responses in cereal crops [47]. As this is the first sorghum secretome study under combined osmotic and heat stress, our results lay a foundation for future investigations on the composition and function of secreted proteins in plants growing in hot and dry environments. Some of the stress-responsive proteins identified in this study, such as proteases and their inhibitors, antioxidant enzymes, and chaperones, may be potential targets for developing stress-resilient crops. Our working hypothesis is that sorghum cells exposed to individual or combined osmotic and heat stresses, alter cellular metabolism and secrete common and unique proteins to cope with the prevailing stressful environments and promote plant survival.

## 2. Results and Discussion

With a progressively warming global climate, combined drought and heat stress will likely cause more significant losses in crop yield than the individual stresses of drought and heat [3,4]. As such, understanding the effects of combined drought and heat stress in plants is an emerging focus for many researchers worldwide. Proteomic studies of the response to combined drought and heat stress have to date focused on the intracellular leaf proteome [31,32,33,34,35], with no published work on secreted proteins of the extracellular matrix (ECM). However, the secretome plays essential roles in cell growth, signalling, communication, and defence responses against biotic and abiotic stresses [41,42,48,49]. Therefore, this study aimed to investigate the effects of combined osmotic and heat stress on protein secretion in sorghum cell suspension cultures. Results obtained from such a study could be useful in identifying potential targets for developing stress-resilient crops. We used sorghum cell suspension cultures in line with our longstanding research interests to understand the influence of abiotic stresses on protein secretion in plants [50,51]. The stresses applied in this study consisted of individual treatments or a combination of osmotic stress [50] and heat stress at 40 °C [51] used in previous studies.

### 2.1. Osmotic Stress, Heat, and Their Combination Exert Differential Effects on Sorghum Cell Metabolic Activity

**Figure 1:**
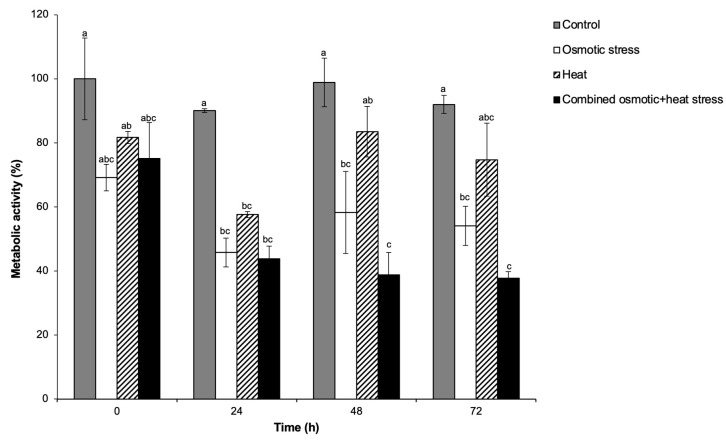
Metabolic activity of ICSB338 sorghum cell cultures under single and combined stresses. ICSB338 sorghum cell cultures were exposed to osmotic stress (400 mM sorbitol at 27 °C), heat (40 °C), and a combination of osmotic and heat stress (400 mM sorbitol at 40 °C) for 72 h. Control cell cultures were maintained at 27 °C for the duration of the experiment. Cell aliquots were taken at the indicated time-points for assessment of the metabolic activity using the MTT (3-(4,5-dimethylthiazolyl2-2,5-dipenyltetrazolium) assay. Data presented as the mean ± SE (*n* = 3). Different letters indicate significant difference between means at (*p* ≤ 0.05) according to ANOVA and Tukey-Kramer test.

Analysis of metabolic activity revealed a significant dip 24 h after exposure to all three treatments: osmotic stress, heat stress, and their combination relative to the control (Figure 1). However, the heat-stressed cells regained their full viability to levels indistinguishable from the controls at 48 and 72 h. This implies that the 40 °C heat threshold is not lethal to ICSB338 sorghum cells, a result previously reported by Ngcala et al. [51]. The osmotically stressed cells and those exposed to combined osmotic and heat stress did not fully recover from the stress within the 72-h treatment period when compared to the control. Furthermore, sorghum cell cultures exposed to the combined stress had the least metabolic activity at 48 and 72 h post-treatment (Figure 1), illustrating the heightened negative impact of the stress combination on ICSB338 sorghum cells. The diminished metabolic activity is perhaps the basis for the growth and yield penalty often associated with the combined drought and heat stress seen in the field. Other studies have also shown that combinations of heat and water deficit are more disruptive to normal plant cell metabolism when compared to individual stresses [52]. Overall, our results suggest that combined osmotic and heat stress has greater detrimental effects on cellular metabolism than the single stresses applied individually.

### 2.2. Proline and Glycine Betaine Accumulation Is Not Universal across Osmotic Stress, Heat Stress, or Combined Stress

**Figure 2:**
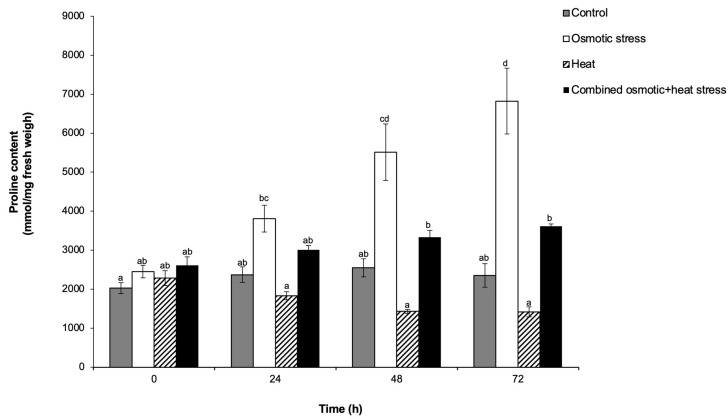
Proline content of ICSB338 sorghum cell cultures under individual and combined stresses. ICSB338 sorghum cell cultures were exposed to osmotic stress (400 mM sorbitol at 27 °C), heat (40 °C), and a combination of osmotic and heat stress (400 mM sorbitol at 40 °C) for 72 h. Control cell cultures were maintained at 27 °C for the duration of the experiment. Cell aliquots were taken at 0, 24, 48, and 72 h for proline content analysis using hydrophilic interaction chromatography and liquid chromatography–mass spectrometry. Data are presented as the mean ± SE (*n* = 3). Different letters indicate significant differences between means at (*p* ≤ 0.05) according to ANOVA and Tukey-Kramer test.

Plants also respond to abiotic stresses by accumulating diverse metabolites with signalling, osmoregulatory, and protective functions [53,54]. Proline and glycine betaine have been studied extensively, and their accumulation patterns in many plant species under a range of abiotic stresses are documented [53,54]. Nevertheless, as reviewed by Ngara and Chivasa [22], increased proline accumulation is not necessarily universal across treatments of drought, heat, and their combination, even within a single plant species. In this study, we only observed a statistically significant increase in proline levels in osmotically stressed sorghum cell cultures at 48 and 72 h compared to the control (Figure 2). Although the proline content of the heat-stressed cells showed a declining trend throughout the 72 h of treatment, it was below the threshold of statistical significance when compared to the controls. Likewise, the combined osmotic and heat stress had a marginal increase in proline accumulation, though it was also not statistically different from the controls. However, heat stress alone moderately lowered proline content below the combined osmotic and heat stress at 48 and 72 h (Figure 2). Similar findings were reported in Arabidopsis (*Arabidopsis thaliana*) leaves, where proline accumulated to high levels under drought conditions but was inhibited under heat stress alone and when drought was combined with heat stress [25].

While the reasons behind such inhibitory effects of heat stress or combined heat and drought stress are not clear, proline and/or its intermediate metabolites may be toxic to some plant species under these conditions [25]. In such cases, the osmoregulatory and protective functions of proline may be assumed by soluble sugars [25]. In other studies, drought, heat, and combined drought and heat stresses resulted in increased accumulation of proline in leaves of wheat (*T. aestivum*) [55], tomato (*Solanum lycopersicum*) [52] and soybean (*G. max*) [27] plants. However, the highest proline content was reported under heat stress in the soybean study [27] and combined drought and heat stress conditions in wheat [55] and tomato [52]. In the current study, levels of glycine betaine remained unchanged throughout the 72 h of treatment with osmotic stress, heat, and their combination of stress (Figure S1). This possibly indicates that glycine betaine does not play a significant role in the stress-adaptive responses of ICSB338 sorghum cell cultures to these treatment conditions. In contrast, Goche et al. [56] reported an increased accumulation of glycine betaine in SA1441 and ICSB338 sorghum leaves and roots under drought stress. It is, however, unclear why glycine betaine accumulates in drought-stressed sorghum leaf and root tissues [56] but not in sorghum cell suspension cultures exposed to sorbitol-induced osmotic stress (Figure S1). Perhaps these contrasting results are due to differences between whole plant and cell culture experimental systems. Contrary to our results, glycine betaine accumulated in watermelon (*Citrullus lanatus*) cell suspension cultures exposed to 100 mM mannitol-induced osmotic stress [57]. The authors also showed that exogenous glycine betaine alleviated cell growth retardation caused by the osmotic stress [57]. Nonetheless, our results suggest that glycine betaine and proline accumulation is not a universal response in ICSB338 sorghum cell suspension cultures under osmotic stress, heat, and the combined stress. Future metabolomics studies of ICSB338 sorghum cell cultures should investigate possible metabolite replacements of these two organic compounds under similar treatments. Such investigations could contribute insights into the range of stress-responsive osmolytes under single and combined stresses.

### 2.3. Combined Osmotic and Heat Stress Modulates Protein Secretion in Sorghum Cell Cultures

The growth medium of cell suspension cultures is widely used as an in vitro source of secreted proteins [41,42,58]. In this study, secreted proteins were extracted from the growth medium of control cells and those treated with combined osmotic and heat stress 72 h following treatment. The 72-h time point was selected in line with previous sorghum secretome studies [50,51,59] and the observed changes in cell metabolism upon stress treatment (Figure 1 and Figure 2). Extracted ECM proteins were labelled using the isobaric tags for relative and absolute quantification (iTRAQ) method and analysed by liquid chromatography-tandem mass spectrometry (LC-MS/MS). After cleaning up the MS/MS data, 459 secreted proteins were positively identified with at least two peptides and the full peptide information is provided in Table S2. The minimum threshold of two sequenced peptides was selected to increase the confidence in protein identification. Of these 459 proteins, 117 were responsive to combined osmotic and heat stress according to a Student’s *t*-test at *p* ≤ 0.05 (Table S3). Protein accession numbers of the differentially expressed protein (Table S3) and their corresponding amino acid sequences from the UniProt database were used in bioinformatics analyses to update the protein names, predict the presence of signal peptides, and assign GO terms and protein families (Table S4). Due to the extensive list of the differentially expressed proteins identified in this study (Table S4), we show a subset of these proteins (Table 1) filtered using a probability cutoff threshold of *p* ≤ 0.01 for the significance of differential expression between the control and stressed sample. However, the entire protein list (Table S4) is used in the discussion of results.

A total of 112 (~96%) of the 117 differentially expressed proteins were annotated with protein names on the UniProt database as of 31 March 2024 (Table S4). This is a notable improvement in the current state of sorghum proteome annotations compared to the 26–34% observed in our previous sorghum proteomics studies of roots [56] and cell suspension cultures [50,51,59]. Such developments in sorghum proteome database annotations will greatly support computational and experimental studies aimed at understanding the function of stress-responsive proteins and, hence, the molecular basis of stress adaptation in this crop.

### 2.4. Majority of Differentially Expressed Proteins Use the Classical Secretory Pathway for Extracellular Localization

**Figure 3:**
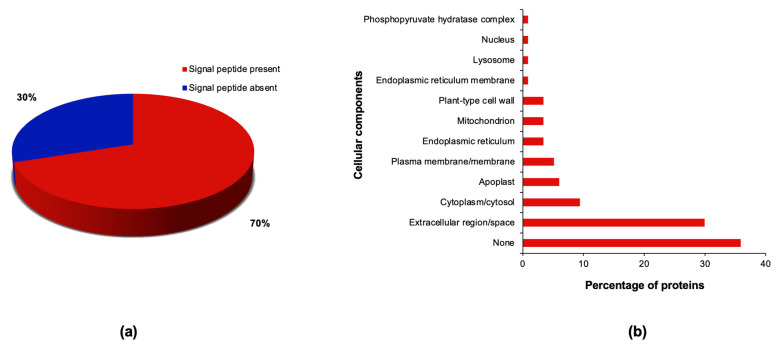
Predictions of signal peptide and cellular locations of the combined osmotic and heat stress-responsive sorghum secretome. (**a**) Signal peptide predictions using SignalP 6.0. (**b**) Gene Ontology terms for cellular components retrieved from the UniProt database.

Protein localisation is a key aspect of protein function since mislocalised proteins will not have access to their intended substrates or the structural scaffolding for complex formation. The N-terminal signal peptide prediction results revealed that a higher fraction of the combined stress-responsive proteins (~70%) had signal peptides (Figure 3a). Previous proteomics studies also identified an abundance of signal peptide-containing proteins in secreted protein fractions of sorghum cell cultures [50,51,59]. N-terminal signal peptides direct proteins to the classical secretory pathway, which involves the endoplasmic reticulum (ER), Golgi apparatus and the Trans Golgi network [60]. This signal peptide tagging system facilitates the proper localisation of proteins for optimum cell functioning [61]. Conversely, other secreted proteins, known as leaderless proteins, lack signal peptide sequences and are probably translocated out of the cell by unconventional secretory pathways [60,62]. Examples of signal peptide-containing proteins identified in the current study include several members of glucoside hydrolases, plant peroxidases, thaumatins, germins, aspartic peptidases, cystatins, protein exordium-like, multicopper oxidases and GDSL lipase/esterases (Table S4). On the other hand, those lacking signal peptides include superoxide dismutase (SOD), malate dehydrogenase, glyceraldehyde-3-phosphate dehydrogenase and other members of glucoside hydrolases and plant peroxidases (Table S4). Similar proteins with and without signal peptides have also been identified in secretome studies of sorghum under osmotic stress [50], heat [51], and exogenous ABA treatment [59] and various other plant species under biotic and abiotic stresses [41,63]. Our results suggest that sorghum secreted proteins may or may not contain signal peptides, further alluding to the existence of diverse protein secretory pathways in plants. However, subcellular localization experiments are required to validate the in vivo locations of these proteins under control and stress conditions.

Gene Ontology (GO) terms are used to functionally annotate proteomes and better understand the biological significance of proteomics data under defined experimental conditions [64,65,66]. Moreover, subcellular localization of proteins is also annotated by most GO tools freely available online. We retrieved GO terms for the 117 stress-responsive proteins to assist with their functional annotation and subcellular localization (Table S4). The results revealed that 39% of the 117 stress-responsive proteins were associated with extracellular locations, 25% with intracellular locations, and 36% had unknown cellular components (Figure 3b). This large fraction of sorghum secreted proteins whose cellular components are unknown calls for improvements in GO annotations through experimentally derived protein localization data, or inferences from phylogenetic studies and homology-based computational predictions [66]. Of the 42 proteins without GO annotations for cellular components, 32 (76%) had predictable signal peptides (Table S4), thus pointing to their extracellular localization. These results also contribute to an increase in the number of proteins with potential extracellular locations in this study. However, further improvements in GO annotation of sorghum proteins in genomic databases are still required. Perhaps this will come from inevitable developments in bioinformatic tools capable of highly accurate automation of protein function, structure, and localization prediction.

The extracellular-related locations included terms such as extracellular region, extracellular space, apoplast, and plant-type cell wall (Figure 3b), and most of the associated proteins had predictable signal peptides except for a few leaderless glycoside hydrolases, peroxidases, and SODs (Table S4). Conversely, the intracellular-related locations included GO terms such as cytoplasm, cytosol, plasma membrane/membrane, and organelles/organelle-related structures such as the ER, ER membrane, mitochondrion, lysosome, and nucleus (Figure 3b). As expected, most proteins associated with intracellular locations lacked predictable signal peptides expect for five membrane-related proteins and all ER-related proteins (Table S4). While the ER is located inside the cell, it is an integral component of the classical secretory pathway, where proteins are both co- and post-translationally modified prior to secretion [60,62]. Endoplasmic reticulum proteins are also imported into the ER lumen via N-terminal signal peptides [61]. Still, our results highlight the need for further subcellular localization studies to validate the locations of the stress-responsive proteins. In addition, the extent of protein moonlighting [67] in plants under stress ought to be investigated, as some intracellular proteins may have different functions outside the cell.

### 2.5. Putative Functional Groups of the Combined Osmotic and Heat Stress-Responsive Secretome

**Figure 4:**
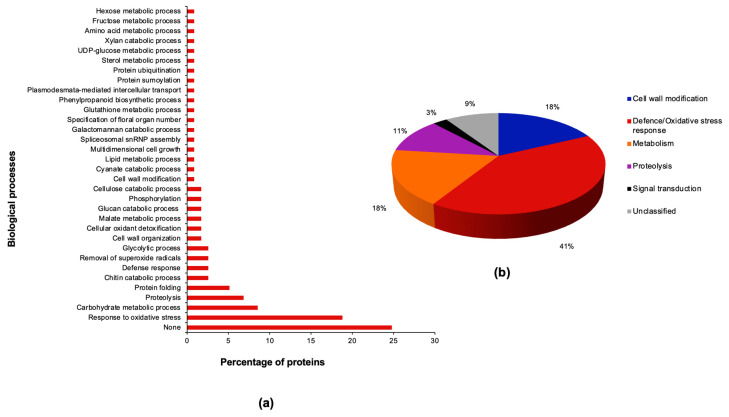
Biological processes and functional groupings of combined osmotic and heat stress-responsive sorghum secretome. (**a**) Gene Ontology terms for biological processes were retrieved from the UniProt database. (**b**) Pie chart showing the functional distribution of the stress-responsive secretome.

We used the protein family names in combination with GO terms for biological process and molecular function to functionally group the 117 stress-responsive secreted proteins (Table S4; Figure 4a,b). The results revealed a large fraction of the proteins were involved in defence and oxidative stress response (41%), followed by cell wall modification (18%), metabolism (18%), proteolysis (11%) and signal transduction (3%) (Figure 4a,b). However, 9% of the proteins remained unclassified due to their limited GO annotations (Table S4). Summaries of the biological processes (Figure 4a) and molecular functions (Figure S2) of the stress-responsive proteins highlight the diverse functional roles of secreted proteins under stress. Below is a brief description and discussion of the main trends observed in each functional group.

#### 2.5.1. Defence/Oxidative Stress Response

Of the 117 stress-responsive proteins, 48 (41%) are involved in biological processes broadly related to plant defence and oxidative stress response and belong to 14 protein families (Table S4; Figure 4b). Most of these proteins (26, equivalent to 54%) were upregulated, while the rest were downregulated. Extracellular plant peroxidases dominated this functional group with 22 proteins, of which 20 were downregulated. The only other downregulated proteins in this functional group were a chitinase with accession C5YBE9 and a small nuclear ribonucleoprotein Sm D3 protein with accession C5WRK4. Conversely, the upregulated protein families are related to antioxidant activities (e.g., plant peroxidases, SODs, glutathione-S-transferase (GST), dehydroascorbate reductase and peroxiredoxin-5-like), defence-related processes (e.g., chitinases, thaumatin, dirigent and dienelactone hydrolases), and protein folding/chaperone activities (e.g., calreticulin, protein disulfide isomerases, and heat shock protein 70 (HSP70)). Due to the wide diversity of protein families identified in this functional group (Table S4), our discussion is focused on major trends in the subcategory of defence/antioxidant response.

Drought and heat stresses induce the overproduction and accumulation of reactive oxygen species (ROS), which may cause oxidative stress damage to cell structures [1,2]. Various enzymes involved in the generation and/or detoxification of ROS were identified in this study, including germins, plant peroxidases, SODs, GST, dehydroascorbate reductase and peroxiredoxin-5-like (Table S4). Plant peroxidases are encoded by a large multigene family; therefore, these proteins may possess varying substrate specificities and physiological functions in plants [68]. Depending on their substrates, plant peroxidases may oxidize molecules by consuming hydrogen peroxide or generate ROS such as superoxide or hydroxyl radicals [69,70]. As such, peroxidases are involved in several physiological and defence processes, including lignification, suberization, cross-linking of cell wall proteins, senescence, and responses to biotic and abiotic stresses [68,69,70]. With such diverse functional roles of plant peroxidases, it is unclear why the combined stress treatment resulted in a massive downregulation of 20 of the 22 secretory peroxidases identified in this study (Table S4). These results (Table S4) are in stark contrast with those of our previous sorghum secretome study under sorbitol-induced osmotic stress, where 20 of the 22 identified plant peroxidase family proteins were upregulated [50]. In another sorghum secretome study under heat stress, 10 of the 17 stress-responsive peroxidases were downregulated [51].

To demonstrate the dynamics in accumulation patterns of the identified peroxidases, we compared the iTRAQ data of the current study (Table S4) with that of our previously published sorghum secretome studies under osmotic [50] and heat [51] stresses (Table 2). Of the 22 differentially expressed peroxidases identified in the current study, eight accessions, C5Z475, C5X5K6, A0A1B6QFT1, C5XL59, C5XIY1, C5YQ75, C6JSB7 and C5YQ75, were also responsive to the individual treatments of osmotic stress and heat. Accessions C5Y360, C5X3C1, A0A1B6QGB6 and C5YZJ2 were only responsive towards osmotic and combined stress treatments. Similarly, peroxidase proteins A0A1W0W7I8, C5Z469, C5X040, C5XYY5, C5Z0N8 and A0A1W0W7T8 were responsive to heat stress and the combined stress, while C5WYQ4, C5X0X1, C5YHR8 and C5X3C6 were unique to combined stress. Since a subset of the combined stress-responsive peroxidases from the current study (Table S4) were “not detected” in data derived from previous secretome studies (Table 2) under individual treatments of osmotic [50] and heat [51] stress, we proposed a meta-analysis using raw iTRAQ data from various studies to ascertain the shared and unique stress-responsive proteins between single and combined stresses. While we did not test statistical differences in fold changes between the three studies, the upward and downward trends in protein regulation and magnitudes of change varied considerably between the studies (please refer to supplementary protein Tables of each study). From our observations, however, the heat stress results seemed more like that of the combined stress, as nine of the 22 peroxidases were downregulated by both stresses (Table 2). Nevertheless, the implications of such common and unique protein expression patterns in the sorghum ECM under osmotic stress, heat and their combination require further investigations.

Apart from the ROS-generating peroxidase, we identified an upregulated germin-like protein with accession C5XHX2 (Table S4). Interestingly, this protein isoform was also highly upregulated in the sorghum secretome following exposure to individual treatments of osmotic stress [50] and heat [51]. Germins possess oxalate oxidase activity and produce hydrogen peroxide from the degradation of oxalate [71,72]. In a recent review, Govindan et al. [73] summarized the critical roles of germin-like proteins in biotic and abiotic stress responses of various crops and their potential as candidates for crop improvement strategies. The authors also highlighted the strong upregulation of these proteins under pathogen infection, herbivore-induced damage, drought, desiccation, salt, cold, heat and various hormonal treatments. Similarly, the increased accumulation of the germin-like protein with accession C5XHX2, in the current study and under osmotic stress [50] and heat [51] suggests its central role in the adaptive responses of sorghum to abiotic stress.

Apart from the ROS-generating germins and peroxidases mentioned above, we also identified several ROS-detoxifying enzymes in the sorghum ECM (Table S4). These included three SOD isoforms, GST, peroxiredoxin, and dehydrogenase reductase and all were highly upregulated with fold changes ranging between 1.9 and 3.2 (Table S4). SODs are known to dismutase superoxide radicals to hydrogen peroxide [74], while GST detoxifies ROS and other toxic metabolites by conjugation with glutathione, producing less toxic compounds for further processing [75,76]. GSTs are also crucial in detoxifying end-products of membrane lipid peroxidation, which are highly cytotoxic [75]. At low levels, however, ROS, such as hydrogen peroxide, may act as signalling molecules for the increased expression of genes and proteins with protective roles against various stresses [77]. Therefore, maintaining cellular redox homeostasis under stress conditions prevents oxidative stress damage while facilitating ROS signalling in plants. Accordingly, our results suggest that combined osmotic and heat stress triggers oxidative stress in the sorghum apoplast, which is mitigated by an array of antioxidant enzymes. Other secretome studies have also identified increased accumulation of ROS-scavenging enzymes in response to osmotic [50] dehydration [78,79] and heat [51,80] stresses. However, the physiological and molecular contributions of these ROS-metabolizing enzymes towards combined stress adaptive responses require further characterization using transgenic plants [73] and by measuring specific antioxidant enzymatic activities in cell culture media [81] under stress.

#### 2.5.2. Cell Wall Modification

Our results indicated that 21 (18%) of the differentially expressed proteins were related to cell wall modification (Table S4; Figure 4b). This functional group was dominated by members of glycoside/glycosyl hydrolases that specifically metabolise cellulose, glucose, glucans, xylan, galactomannan, galactosides, pectins and xyloglucans (Table S4). Of these proteins, 13 (62%) were downregulated, while the rest were upregulated. Examples of the downregulated proteins include beta-D-xylosidase, beta-glucosidases, beta-glucuronidase, endoglucanases, alpha-galactosidases, xyloglucan endotransglucosylase/hydrolase (XET), pectin methylation modulator, pectinesterase, expansin and a fasciclin-like arabinogalactan protein. Conversely, the upregulated proteins included a Pla a 1-like protein with pectinesterase inhibitor activity, glucan endo-1,3-beta-D-glucosidase, beta-glucosidases, beta-fructofuranosidase and UTP—glucose-1-phosphate uridylyltransferase (Table S4). While the significance of this differential expression of cell-wall remodelling proteins would need further investigation, to an extent, our results suggest that sorghum cells under combined osmotic and heat stress downregulate secreted proteins associated with cell loosening, expansion, and cell growth such as expansins and XETs [82,83]. These results contrast those of our previous sorghum secretome study under osmotic stress, which implied that osmotic stress encouraged increased accumulation of expansins and XETs [50], possibly to increase cell growth for enhanced water foraging capacity. Various other cell wall modifying proteins were also identified in secretome studies under heat [51,80], cold [84,85], UV-B radiation [86] and dehydration [78,79] stresses. By performing time-course experiments under dehydration stress, Bhushan et al. [78] and Pandey et al. [79] further demonstrated complex temporal dynamics of protein expression under stress. Such results also highlight the need for more comprehensive experimental designs to gain deeper insights into plant responses to combined stresses.

In addition, our current proteomic results suggest that the combined stress affects pectin modification by downregulating a pectinesterase and a pectinesterase methylation modulator while upregulating a pectinesterase inhibitor (Table S4). Pectin is a complex polysaccharide that forms an essential component of plant cell walls. Pectins are modified by pectin methylesterases, whose activities are regulated by pectin methylesterase inhibitors [87,88,89]. Pectinesterases modify cell wall pectin via demethylesterification, a process determining whether the cell wall loosens or stiffens [87,90,91]. Due to the multiple roles of pectinesterases in plants [90,91], further investigations are required to understand their effects on cell wall structure under combined osmotic and heat stress. Overall, our proteomic results demonstrate the complex structural composition of plant cell walls and the dynamic nature of cell wall remodelling processes under combined osmotic and heat stress, which may be similar or different under the individual stresses of osmotic stress [50] and heat [51].

#### 2.5.3. Proteolysis

We identified 13 (11%) proteolysis-related proteins that were responsive to the combined stress treatment (Table S4, Figure 4b). Aspartic peptidase A1 family members dominated this functional group with eight proteins, of which seven were downregulated. The rest of the proteins were upregulated with high fold changes of 1.7–2.8 and belonged to the ubiquitin and ubiquitin-like protein family and protease inhibitor families of the cystatin and proteinase inhibitor I3, Kunitz types (Table S4). Proteases are a large group of protein-degrading enzymes with diverse biological functions during plant development and in response to biotic and abiotic stresses [92,93,94]. Catalytic activities of proteases are partly regulated by protease inhibitors, whose accumulation may also be modulated by stress [94,95]. Although a wide range of apoplastic proteases and their inhibitors have been identified in plant secretomes subjected to water deficits [50], heat [51], cold/freezing stress [84,96], UV-B radiation [86] and pathogen infection [97,98], we observed a noteworthy trend in the current study: a strong downregulation of aspartic peptidases and an upregulation of protease inhibitors and ubiquitin-related proteins (Table S4).

Functionally, aspartic peptidase A1 family proteins mobilise nitrogen sources during seed germination, leaf senescence and programmed cell death [99,100]. During stress response, these proteases may regulate protein turnover and mobilise amino acid pools for the synthesis of stress-responsive proteins. In contrast, extracellular aspartic proteases may prevent the over-accumulation of pathogenesis-related proteins [99]. Therefore, the remarkable downregulation of secreted aspartic proteases observed in this study (Table S4) could be a protective mechanism against the degradation of stress-responsive proteins in the ECM. This reasoning is partly supported by the observed upregulation of two proteinase inhibitors (Table S4), which inhibit cysteine- and serine-type proteases, respectively [94,95]. Increased accumulation and activity of protease inhibitors in plants under abiotic stresses such as drought are often associated with enhanced stress tolerance [101]. Although proteolysis is essential for maintaining cell protein homeostasis, unregulated protein degradation may be lethal to biological systems [92]. The upregulated ubiquitin and ubiquitin-like proteins identified in this study (Table S4) are potential components of the ubiquitin–proteasome system (UPS), a highly selective protein degradation system in plants [92,102]. Ubiquitin marks proteins for selective degradation by the proteasome, preventing random proteolytic activities in cells while maintaining protein homeostasis in response to stress [102]. Our results suggest some tight regulatory control of proteolytic activity in the sorghum ECM under combined heat and osmotic stress. This control mechanism is partly achieved by downregulating aspartic proteases and inhibiting the activities of other proteases while selectively tagging unwanted proteins for degradation via the ubiquitin–proteasome system. Nevertheless, functional validation studies are required to ascertain the biological roles of these proteins in stress response. Various aspartyl protease isoforms were either up or downregulated in secretome of Arabidopsis genotypes exposed to UV-B radiation [86]. The authors also highlighted the difficulty in postulating reasons behind opposite expression trends of members of the same protein family in the same eperimental sample. As with many “omics” data, functional validation studies are required to understand the role of proteolysis-related proteins including aspartic peptidase in combined stress response.

#### 2.5.4. Signal Transduction

The signal transduction functional group comprises three proteins (Table S4; Figure 4b). A protein kinase domain-containing protein was downregulated, while a nucleoside diphosphate kinase and an EF-hand domain-containing protein were upregulated with fold changes of 1.5 and 3.7, respectively (Table S4). Nucleoside diphosphate kinases (NDPKs) are involved in the production of diverse nucleoside triphosphates (NTPs) required for cell signalling, stress response, and synthesis of nucleic acids, lipids, polysaccharides, and proteins [103]. According to Dorin and Rivoal [103], different NDPK isoforms are localized in the cytosol, various organelles, extracellular matrix, or the secretory pathway, a feature that supports their multifunctional roles in plants. The identified NDPK protein with accession C5WRH5 has no predictable signal peptide and its subcellular compartment is not yet known (Table S4). However, its increase in response to combined stress, together with increases of the metabolism-related UTP--glucose-1-phosphate uridylyltransferase (accession A0A1B6QE21, Table S4), suggests a well-coordinated stress response system involving diverse biological processes aimed at improving plant survival under stressful conditions. Some NDPK transcripts and/or proteins are induced by salt, cold, drought, wounding, and pathogen infection [103], thus supporting results of the current study. Lee et al. [104] also observed that tall fescue (*Festuca arundinacea*) transgenic plants, overexpressing an *A. thaliana NDPK2* gene, exhibited greater salt tolerance compared to the wild-type plants, possibly through enhanced antioxidant capacity and stress signalling. However, more studies are required to establish the identified NDPK protein’s subcellular localization, substrate(s), interacting protein partner(s), and function in combined stress response.

Another highly upregulated protein in this functional group was an EF-hand domain-containing protein (accession C5XQS6) with a predicted calcium ion binding molecular function (Table S4). EF-hand domain-containing proteins are calcium-binding proteins that sense stress-induced increases in cytosolic calcium levels [105,106]. Under abiotic stress conditions, EF-hand domain-containing proteins participate in calcium signalling upstream of signal transduction cascades that activate alterations in gene expression and cell metabolism for survival [106]. Transcripts of calcium sensors with EF-hand domains such as calmodulin, calmodulin-like proteins, calcium-dependent protein kinases and calcineurin-B-like proteins are also modulated by drought, heat, combined drought and heat, salt, and fungal stresses [107]. Therefore, the observed upregulation of the EF-hand domain-containing protein (Table S4) suggests that calcium signalling is activated in the sorghum ECM under combined osmotic and heat stress. The involvement of calcium signalling during plant response to abiotic stress has been known for decades [108]. A secretome study of Arabidopsis cells under salicylic acid treatment also identified a leaderless calmodulin as one of the most upregulated proteins [109]. However, the functions of extracellular EF-hand domain-containing proteins, including extracellular calmodulin, in stress adaptation require further investigation.

Various signalling pathways related to drought, cold, heat, salinity, and oxidative stress response are associated with reversible protein phosphorylation events, catalyzed by diverse protein kinases [110,111]. Under these environmental conditions, protein phosphorylation acts as a molecular switch that activates regulatory proteins such as transcription factors and various other proteins with structural and protective functions for greater plant survival [111,112]. Kinases are highly specific on their targets and mainly act on serine, threonine, and tyrosine residues and to a lesser extent on histidine and aspartate residues [112]. We identified a signal peptide-containing plasma membrane-localized putative protein kinase (accession C5WPY7), among downregulated stress-responsive proteins (Table S4). Plasma membrane-bound serine/threonine protein kinases regulate various processes during plant growth and in response to environmental stresses by perceiving stress signals via their extracellular domains and relaying information to target proteins via their cytoplasmic kinase domains [113]. These membrane-bound kinases are part of a large gene family of receptor-like kinases [113], and their regulation during stress responses may be dynamic in space and time. However, further genetic studies are required to explore its possible role in stress response.

#### 2.5.5. Other Functional Groupings

About 21 (18%) and 11 (9%) of the differentially expressed proteins were assigned to the metabolism and unclassified functional groups, respectively. In the metabolism-related group, 12 (57%) were downregulated, while the rest were upregulated. Most of the downregulated proteins were associated with the metabolism of sugars (e.g., glyceraldehyde-3-phosphate dehydrogenase, phosphoglycerate kinase, aldose 1-epimerase, and fructokinase), lipids (e.g., GDSL lipases/esterase like and glycerophosphodiester phosphodiesterase), sterols (e.g., Cytochrome P450), and amino acids (e.g., aspartate aminotransferase). In contrast, upregulated proteins included malate dehydrogenases, cyanate hydratase, phosphopyruvate dehydrogenases, enoyl reductase (ER) domain-containing protein and dihydrolipoamide dehydrogenases. The unclassified proteins have minimal GO annotations (Table S4), and thus, it is difficult to infer their roles in sorghum response to the combined stress. Nevertheless, proteins such as purple acid phosphatase, protein exordium-like, protein of unknown functions and leucine-rich repeat domain-containing proteins were identified, and most have also been identified in other sorghum secretome studies in response to osmotic stress [50], heat [51] or ABA treatment [59]. Amongst these unclassified proteins, a leucine-rich repeat domain-containing protein with accession C5Y2R8 was the most upregulated protein with a fold change of 3.8. In contrast another leucine-rich repeat domain-containing protein with accession C5XBP7 was the most downregulated protein. These results suggest that different protein isoforms of the same family might exhibit differential responses to a given stress treatment, probably due to their varying biochemical activities and roles in stress adaptation.

### 2.6. qPCR Analysis of Target Genes Following Combined Osmotic and Heat Stress Treatment

**Figure 5:**
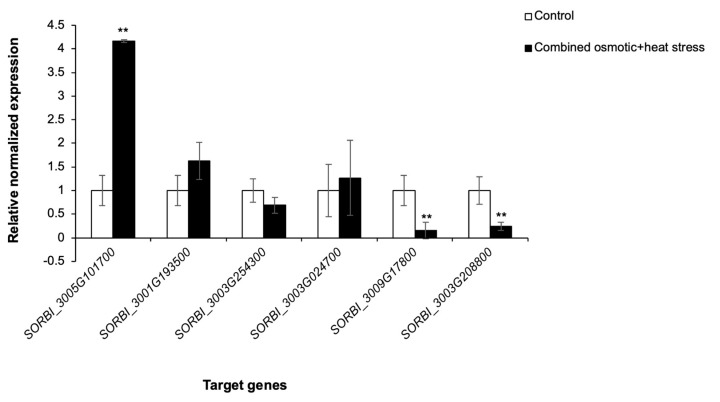
Gene expression of ICSB338 sorghum cell cultures in response to combined osmotic and heat stress. ICSB338 sorghum cell cultures were exposed to a combination of osmotic and heat stress (400 mM sorbitol at 40 °C) for 72 h, while control cell cultures were maintained at 27 °C for the duration of the experiment. Cell aliquots were harvested at 72 h and gene expression analysis was performed using qPCR. Bars represent mean ± SE (*n* = 3). ** indicated significance at *p* ≤ 0.01 using a Student’s *t*-test.

We validated the proteome data (Table S4) by analyzing gene expression profiles of a few targets using qPCR (Figure 5). The *DIRIGENT* gene (*SORBI_3005G101700*) was significantly upregulated in response to the combined stress, while *GLUTATHIONE DEHYDROGENASE *(*ASCORBATE*) (*SORBI_3009G017800*) and *ASPARTIC PEPTIDASE* (*SORBI_3003G208800*) were downregulated (Figure 4). The upward and downward trend in expression of the *DIRIGENT* and *ASPARTIC PEPTIDASE* genes, respectively, correlated with that of their proteins, as observed in the proteomics data (Table S4). These results indicate that the abundances of some stress-responsive proteins are transcriptionally regulated. However, there were no observed changes in the expression of *HSP70* (*SORBI_3001G193500*),* GLUTAREDOXIN-DEPENDENT PEROXIREDOXIN* (*SORBI_3003G254300*) and *PEROXIDASE *(*SORBI_3003G024700*) (Figure 5). This suggests that post-transcriptional regulation underpins the protein expression data (Table S4) or that any transcriptional changes are transient.

## 3. Materials and Methods

### 3.1. Stress Treatments of Sorghum Cell Suspension Cultures

ICSB338 sorghum cell suspension cultures were used in this study. The cell cultures were initiated from friable callus, subcultured and maintained as described previously [114]. All stress treatments were imposed on exponentially growing 8-day-old cell cultures [114]. Osmotic stress was applied using 400 mM sorbitol (S3889, Sigma Aldrich, Saint Louis, MO, USA) at 27 °C [50], and heat stress was imposed at 40 °C without sorbitol [51] in line with our previous research studies. Combined osmotic and heat stress was inflicted using 400 mM sorbitol at 40 °C, while control cells were maintained at 27 °C [50,51]. Four biological replicate cell cultures were generated for all four treatment groups and maintained for 72 h, with samples taken at intervals for biochemical, proteome and gene expression analyses.

### 3.2. Analysis of Cell Metabolic Activity and Cellular Osmolyte Content

The metabolic activity and osmolyte content of sorghum cell cultures were assayed over the 72-h treatment period across all four treatment groups: control, osmotic stress, heat, and combined osmotic and heat stress. The metabolic activity was used to evaluate effect of the imposed stresses on the viability of sorghum cell cultures. Likewise, we analysed the osmolyte content of sorghum cells to investigate the imposed stress effects on known biochemical processes involved in stress response. Cell cultures were sampled at 0, 24, 48 and 72 h for cell viability measurements using the MTT (3-(4,5-dimethylthiazolyl2-2,5-dipenyltetrazolium) assay [115], and proline and glycine betaine content analysis using hydrophilic interaction chromatography in tandem with liquid chromatography–mass spectrometry (HILIC LC-MS) [56,116]. The osmolyte content was analysed on a QTRAP 6500 MS (Applied Biosystems Sciex, Foster City, CA, USA) following the detailed protocol described in our previous study [56]. In the current study, 0 h denotes the time prior to stress treatment. All measurements were carried out on four biological replicate cell cultures per treatment group. In addition, to the biological replicates, two technical replicates were included for the MTT assay.

### 3.3. Secreted Protein Extraction, iTRAQ and LC-MS/MS Analyses

Proteomic analysis of the secreted proteins was conducted only for the control and combined stress conditions using the isobaric tags for relative and absolute quantification (iTRAQ) gel-free method. Four biological replicate cell cultures were harvested 72 h after stress treatment, and secreted proteins were extracted from the culture medium as described previously [117]. After protein quantification, 12.5 μg aliquots of each sample were used for iTRAQ labelling. Protocols for trypsin digestion, iTRAQ labelling, sample clean-up, and liquid chromatography-tandem mass spectrometry (LC-MS/MS) are as described in our previous publications [56,59]. LC-MS/MS was performed on a Triple TOF 6600 mass spectrometer (Applied Biosystems Sciex) linked to an Eksigent 425 LC system via a Sciex Nanospray III source (Applied Biosystems Sciex), and mass spectrometer data were acquired using the Applied Biosystems Sciex Analyst TF 1.7.1 instrument control and data processing software [59].

### 3.4. Protein Identification and Bioinformatics Analysis

Protein identification and relative quantification were conducted as described by Goche et al. [56] with minor modification as specified in Muthego et al. [59]. Differentially expressed proteins in response to the combined osmotic and heat stress were statistically analysed using the Student’s *t*-test at *p* ≤ 0.05. The stress-responsive proteins were assigned with Gene Ontology terms using the UniProt database [66] and protein family names using the Interpro [118] and Pfam [119] databases to assist with their theoretical functional classification. Signal peptides were predicted using the SignalP 6.0 server [120] to assess the likelihood of the stress-responsive proteins being secreted via the classical secretory pathway.

### 3.5. Total RNA Extraction and Gene Expression Analysis

Three biological replicate control and combined osmotic and heat-stressed sorghum cell cultures were harvested 72 h post-treatment for RNA extraction. The 72-h time-point was selected to correspond with the harvest time used for the proteome analysis. Total RNA was extracted using the Spectrum Plant Total RNA kit (Sigma) according to the manufacturer’s instructions. A DNase digestion step was performed to remove residual genomic DNA during total RNA extraction using the On-Column DNase 1 Digestion Set (Sigma) following the manufacturer’s instructions. A microgram of total RNA was used for complementary DNA (cDNA) synthesis using the GoScript Reverse Transcriptase System (Promega, Southampton, UK) according to the manufacturer’s instructions. Quantitative reverse transcription-polymerase chain reaction (qRT-PCR) was performed using the SsoAdvanced Universal SYBR Green Supermix kit (Biorad, Hercules, CA, USA) following the manufacturer’s instructions. Reaction mixtures consisted of 10 μL of the SsoAdvanced Universal SYBR Green Supermix, forward and reverse primers at a final concentration of 4 μM each, and 5 μL of the cDNA template diluted 1:50 in a final volume of 20 μL. All reactions were performed on a CFX Connect Real-Time System (Biorad) using the following thermal cycling conditions: initial denaturation at 95 °C for 30 s, followed by 40 cycles of denaturation at 95 °C for 10 s, and annealing/extension and plate read at 60 °C for 30 s, and a melt curve analysis using default settings. The thermal cycling protocol used is as described in the Biorad SsoAdvanced Universal SYBR Green Supermix user manual. Data analysis was conducted using the CFX Maestro software version 4.1.2433.1219 with two reference control sorghum genes, *EIF4a1 Sb04g003390* [121] and an uncharacterised *Sb03g03891033* gene [26]. The gene specific primers were designed using the Primer-BLAST tool [122], which is available in the National Centre for Biotechnology Information database. Primer sequences of all target and reference control genes are listed in Table S1. Target genes were randomly selected from the stress-responsive proteins and consisted of both up and downregulated proteins (Tables S1 and S4).

## 4. Conclusions and Future Perspectives

Much of our knowledge of the plant secretome has been gained from studies of plants exposed to individual stresses [41,42,44,58], yet stress combinations often occur under field conditions [3,4]. With the projections in climate change and global warming scenarios, especially in sub-Saharan Africa, drought and heat stress combinations are becoming the leading cause of crop failure and food insecurity in the region [7,8]. Therefore, more drought and heat-resilient crops are required to meet the global food demand in a changing climate. Without a doubt, understanding plant adaptive responses to combined drought and heat stress is a prerequisite for developing crops that are well adapted to a hotter and drier climate. As our study is the first to report on the effects of combined osmotic and heat stress on the sorghum secretome, it lays a foundation for more investigations in this research field.

We observed that 117 (25%) of the 459 sorghum secreted proteins were responsive to the combined stress and had putative functions in defence/oxidative stress response (41%), followed by cell wall modification (18%), metabolism (18%), proteolysis (11%) and signal transduction (3%). These results highlight the specialised functions of secreted proteins in stress-adaptive responses. Furthermore, our “omics” data of sorghum, a naturally drought and heat-tolerant crop, provide candidate stress-responsive genes and proteins for further in silico and experimental characterisation as we aim to improve the annotation of sorghum genes and proteins. We acknowledge that the identified proteins are members of large multigene families and, thus, potentially participate in a myriad of biological processes depending on their subcellular location and stress conditions; hence, we propose further functional validation studies using transgenic overexpression, knockdown or knockout mutant lines to elucidate the role of some of these proteins in stress response. After that, promising candidate genes and proteins with remarkable influence on plant tolerance to combined drought and heat stress could be used in more extensive breeding programmes to evaluate their influence on the growth and yield parameters of field-grown plants under stress. Since our iTRAQ data show a myriad of secreted proteins with and without predicted signal peptide sequences, subcellular localisation experiments using fluorescent protein tagging methods are encouraged to elucidate the locations of these proteins.

The notable trends observed in the current study expand our knowledge of the effects of stress combinations in the apoplast. For example, the upregulation of antioxidant enzymes such as SODs and GST in the sorghum ECM suggests the need for maintaining cell redox homeostasis in this cell compartment. Nevertheless, it remains unclear why ICSB338 sorghum cells massively downregulate extracellular peroxidases in response to the combined stress, in stark contrast to results observed in the secretome of white sorghum cells under osmotic stress [50]. Since plant peroxidases are members of a large multigene family and are involved in generating and detoxifying ROS, more studies are required to ascertain their specific role in combined stress response. Furthermore, physiological and biochemical studies aimed at identifying and quantifying ROS levels and the degree of membrane damage through lipid peroxidation under control conditions and single and combined stresses could elucidate the magnitude of oxidative stress and its effects in the ECM. Likewise, other studies could assess the biochemical activities of enzymatic and non-enzymatic ROS scavengers in the ECM under similar stress treatments to complement and validate our iTRAQ proteomic data. Another study successfully measured peroxidase activities in the culture medium of black pine (*Pinus nigra*) cell suspension cultures [81] and thus illustrates the feasibility of such experiments.

Plant signalling systems are vital components of stress response pathways, and the roles of proteases in generating signalling peptides in the ECM ought to be further investigated [123]. Generally, the secreted stress-responsive proteins are protected from proteolysis via a strong downregulation of aspartic peptidases and an upregulation of inhibitors of other proteases. Furthermore, the identification of highly upregulated ubiquitin-related proteins suggests that the sorghum ECM environment selectively marks unwanted proteins for degradation via the ubiquitin–proteasome system. We propose further characterisation of various sorghum aspartic peptidases using transgenic plants to elucidate their role in plants subjected to single and combined stresses of heat and drought.

Apart from the proteomic data summarised above, this study also highlights that proline and glycine betaine accumulation is not necessarily universal across treatments of osmotic stress, heat, and their combination. These results will inform molecular plant breeders of the complex dynamics in stress response between single stresses and their combinations and guide future crop improvement programmes for a drier and hotter climate. While the current study used cell suspension cultures as a source of the secreted proteins, which is in line with numerous other studies [41], we acknowledge the pros and cons of this experimental system in secretomics. As discussed in previous reviews [42,58], tissue-specific protein expression profiles may not be evident in cell culture-derived proteomics data. As such, we encourage future studies to utilise in planta experimental systems that promote tissue-specific secretome analyses [41,42,58] under combined stresses. In addition, the levels of stress treatments, such as mild, moderate, and severe drought, could be imposed in comparative studies between plant species and even genotypes with contrasting phenotypes within a species. Such diverse experimental systems could generate comprehensive data on the intra- and interspecies similarities and variations in response to different levels and types of abiotic stress. Indeed, our study forms a foundation for more investigations on the composition and biological roles of the plant secretome under combined osmotic and heat stress and opens more avenues for investigating the molecular basis of stress tolerance in plants.

## Figures and Tables

**Table 1:** List of combined osmotic and heat stress responsive secreted proteins of ICSB338 sorghum cell suspension cultures at 1% significance level.

Accession ^a^	Protein Name ^b^	Ratio ^c^	SD ^d^	*p*-Value ^e^	SP ^f^	Protein Family ^g^
Cell Wall Modification						
C5XYP5	Fibronectin type III-like domain-containing protein	−1.41	0.04	3.31 × 10^−3^	+	Beta-D-xylosidase
A0A1B6QHZ6	Beta-glucosidase	−1.34	0.10	6.33 × 10^−3^	−	Cellulose degradation glycosyl hydrolase 3
C5Z8N0	FAS1 domain-containing protein	−1.57	0.12	7.97 × 10^−3^	+	Fasciclin-like arabinogalactan protein
C5WXC7	Alpha-galactosidase	−1.83	0.05	1.89 × 10^−3^	+	Glycoside hydrolase, family 27
A0A1W0W3H0	Alpha-galactosidase	−1.69	0.02	3.02 × 10^−3^	−	Glycoside hydrolase, family 27
A0A1B6QE21	UTP--glucose-1-phosphate uridylyltransferase	1.62	0.14	3.56 × 10^−4^	−	UTP--glucose-1-phosphate uridylyltransferase
C5XT36	Endoglucanase	−2.11	0.12	7.99 × 10^−3^	+	Glycosyl hydrolase, family 9
A0A1Z5R476	Glucan endo-1,3-beta-D-glucosidase GVI	3.87	0.73	8.10 × 10^−4^	+	Glycoside hydrolase, family 17
C5XIT5	Pectinesterase	−1.56	0.07	7.40 × 10^−3^	+	Pectinesterase family
**Defence/Oxidative Stress Response**						
C5WYQ4	Peroxidase	−1.81	0.08	1.38 × 10^−3^	+	Plant peroxidase
C5X5K6	Peroxidase	−1.49	0.07	1.93 × 10^−5^	+	Plant peroxidase
C5XB39	GH18 domain-containing protein	2.69	0.54	1.95 × 10^−3^	+	Glycoside hydrolase 18 family chitinases
C5XJT8	Protein disulfide-isomerase	2.93	0.73	3.88 × 10^−3^	+	Protein disulfide isomerase
A0A1B6PQR2	Protein disulfide-isomerase	2.08	0.44	6.89 × 10^−3^	+	Protein disulfide isomerase
C5XB38	GH18 domain-containing protein	1.41	0.14	5.14 × 10^−3^	+	Glycoside hydrolase 18 family chitinases
A0A1B6QFT1	Peroxidase	−2.24	0.08	2.57 × 10^−4^	+	Plant peroxidase
C5XL59	Peroxidase	−2.93	0.06	1.62 × 10^−3^	−	Plant peroxidase
C5X3C1	Peroxidase	−2.25	0.08	9.64 × 10^−3^	+	Plant peroxidase
C5X3C6	Peroxidase	−2.09	0.08	1.45 × 10^−3^	+	Plant peroxidase
C5XN52	Thaumatin-like protein	2.00	0.43	7.06 × 10^−3^	+	Thaumatin family
C5YQ75	Peroxidase	−1.57	0.06	1.14 × 10^−3^	+	Plant peroxidase
C5YHR8	Peroxidase	−1.40	0.05	3.56 × 10^−3^	+	Plant peroxidase
C5WVD3	Heat shock 70 kDa protein, mitochondrial	3.99	1.37	9.44 × 10^−3^	−	Heat shock protein 70 family
C5YVR0	Superoxide dismutase	2.78	0.07	1.27 × 10^−6^	−	Manganese/iron superoxide dismutase
C5YYX3	Glutathione dehydrogenase (ascorbate)	2.58	0.23	1.25 × 10^−4^	−	Dehydroascorbate reductases DHAR1/2/3/4
A0A1B6QA33	Calreticulin	1.97	0.20	2.23 × 10^−4^	+	Calreticulin
A0A1B6QG28	Superoxide dismutase [Cu-Zn]	3.26	0.75	2.39 × 10^−3^	−	Superoxide dismutase [Cu-Zn]/superoxide dismutase copper chaperone
A0A1Z5RIL8	Dirigent protein	1.94	0.28	3.53 × 10^−3^	+	Dirigent protein
A0A1B6Q818	Glutathione transferase	1.89	0.33	4.12 × 10^−3^	−	Glutathione-S-transferase
C5YXM1	Dienelactone hydrolase domain-containing protein	1.72	0.30	8.64 × 10^−3^	-	Dienelactone hydrolase family
C5YQ75	Peroxidase	−1.57	0.056	1.14 × 10^−3^	+	Plant peroxidase
**Proteolysis**						
C5Z6U2	Ubiquitin-like domain-containing protein	1.97	0.30	3.34 × 10^−3^	−	Ubiquitin and ubiquitin-like
C5Y675	Peptidase A1 domain-containing protein	−1.59	0.03	2.58 × 10^−3^	+	Aspartic peptidase A1 family
C5XQ74	Peptidase A1 domain-containing protein	−1.66	0.08	2.43 × 10^−3^	+	Aspartic peptidase A1 family
C5X3T4	Peptidase A1 domain-containing protein	−1.84	0.07	3.09 × 10^−5^	+	Aspartic peptidase A1 family
C5XQP2	Peptidase A1 domain-containing protein	−1.39	0.02	8.17 × 10^−3^	+	Aspartic peptidase A1 family
C5XG67	Cystatin domain-containing protein	2.76	0.70	6.17 × 10^−3^	+	Cystatin
A0A1B6P6G7	Aspartic proteinase	−2.00	0.11	7.05 × 10^−3^	+	Aspartic peptidase A1 family
**Metabolism**						
C5XX52	Glyceraldehyde-3-phosphate dehydrogenase	−1.47	0.11	3.62 × 10^−3^	−	Glyceraldehyde-3-phosphate dehydrogenase, type I
A0A194YMV2	Phosphoglycerate kinase	2.12	0.30	9.56 × 10^−4^	−	Phosphoglycerate kinase
C5Y9T3	Aldose 1-epimerase	−1.36	0.06	8.43 × 10^−3^	+	Aldose 1-epimerase
A0A1W0VY92	GDSL esterase/lipase	−1.99	0.04	7.30 × 10^−3^	+	GDSL lipase/esterase-like, plant
C5Z861	Phytocyanin domain-containing protein	−1.48	0.06	4.79 × 10^−3^	+	Phytocyanin-like
C5Z4E5	Esterase	−2.77	0.11	3.64 × 10^−4^	+	GDSL lipase/esterase-like, plant
**Signal Transduction**						
C5WPY7	Protein kinase domain-containing protein	−1.85	0.10	2.13 × 10^−3^	+	Protein tyrosine and serine/threonine kinase
C5XQS6	EF-hand domain-containing protein	3.67	0.13	6.94 × 10^−3^	−	None
**Unclassified**						
C5XBP7	Leucine-rich repeat-containing N-terminal plant-type domain-containing protein	−3.70	0.05	3.06 × 10^−5^	+	Polygalacturonase-inhibiting protein
A0A1Z5R915	Purple acid phosphatase	−2.04	0.04	6.53 × 10^−3^	−	Purple acid phosphatase
C5Z6Y0	Uncharacterized protein	−1.66	0.06	1.11 × 10^−3^	+	Protein exordium-like
C5X4M5	DOMON domain-containing protein	−2.45	0.11	1.46 × 10^−3^	+	Protein of unknown function (DUF568)
C5Y2R8	Leucine-rich repeat-containing N-terminal plant type domain-containing protein	3.81	0.31	8.46 × 10^−6^	+	Leucine-rich repeat-containing N-terminal plant type

^a^ Protein accession numbers obtained from the UniProt database searches against sequences of *Sorghum bicolor* only. ^b^ Protein name retrieved from Uniprot database on 31 March 2024. ^c^ Ratio represents the average fold change (*n* = 4) in response to combined osmotic and heat stress relative to the control. A positive value indicates upregulation, while a negative value indicates down-regulation. ^d^ Standard deviation of the fold changes (*n* = 4). ^e^ Probability value (*p* ≤ 0.01) obtained from a Student’s *t*-test comparing the fold changes between the combined osmotic and heat stress treatment and the control (*n* = 4). ^f^ Signal peptide (SP) prediction results for each protein as determined by the SignalP 6.0 server (https://services.healthtech.dtu.dk/services/SignalP-6.0/) (accessed on 31 March 2024). + indicates the presence of a signal peptide, while − indicates the absence of a signal peptide. ^g^ Family name as predicted using the InterPro (http://www.ebi.ac.uk/interpro/) or Pfam (http://pfam.xfam.org) databases. Both the InterPro and Pfam databases were accessed on 31 March 2024.

**Table 2:** Comparison of protein expression trends of combined stress-responsive sorghum secretory peroxidases with other studies.

Protein Accession	Protein Regulation ^a^		
	Combined Stress ^b^	Osmotic Stress ^c^	Heat Stress ^d^
**Common to all stresses**			
C5Z475	down	up	down
C5X5K6	down	up	down
A0A1B6QFT1	down	up	up
C5XL59	down	down	down
C5XIY1	down	up	up
C5YQ75	down	up	down
C6JSB7	down	up	down
C5YQ75	down	up	down
**Combined stress and Osmotic stress**			
C5Y360	down	up	n.d.
C5X3C1	down	up	n.d.
A0A1B6QGB6	down	up	n.d.
C5YZJ2	down	up	n.d.
**Combined stress and Heat stress**			
A0A1W0W7I8	down	n.d.	up
C5Z469	down	n.d.	down
C5X040	up	n.d.	up
C5XYY5	down	n.d.	down
C5Z0N8	down	n.d.	up
A0A1W0W7T8	down	n.d.	down
**Combined stress**			
C5WYQ4	down	n.d.	n.d.
C5X0X1	up	n.d.	n.d.
C5YHR8	down	n.d.	n.d.
C5X3C6	down	n.d.	n.d.

^a^ Protein regulation patterns of each protein as given in the supplementary data files of the respective studies. “Down” in red font represents downregulated proteins, while “up” in black font represents upregulated proteins. n.d. means the protein was “not detected” amongst the differentially expressed proteins of the respective study. ^b^ Results from the current study on combined osmotic and heat stress. ^c^ Results from a published sorghum secretome study under osmotic stress [50] ^d^ Results from a published sorghum secretome study under heat stress [51].

## Data Availability

The datasets generated and/or analysed during this study are available from the corresponding author on request.

## Supplementary Materials

The following supporting information can be downloaded at https://www.mdpi.com/article/10.3390/plants13131874/s1, Figure S1: Glycine betaine content analysis of ICSB338 sorghum cell cultures under osmotic stress, heat and combined osmotic and heat stress; Figure S2: Molecular functions of the sorghum secretome in response to combined osmotic and heat stress. Table S1: List of sorghum primer sequences used in gene expression analysis; Table S2: List of secreted proteins identified in ICSB338 sorghum cell suspension cultures; Table S3: List of sorghum secreted proteins differentially expressed in response to combined osmotic and heat stress; Table S4: List of combined osmotic and heat stress-responsive secreted proteins of ICSB338 sorghum cell suspension cultures at 5% significance level.
