# Supplementary material for: Regulation of Proline Accumulation and Protein Secretion in Sorghum under Combined Osmotic and Heat Stress
Source: Plants (Basel). 2024 Jul 6;13(13):1874. doi: 10.3390/plants13131874 (PMC11244414; doi:10.3390/plants13131874)
Supplement: Supplementary file 1 [file plants-13-01874-s001.zip › Table S1.pdf]

Table S1. List of sorghum primer sequences used in gene expression analysis.

**Accession**	**Gene ID**	**Protein family name**	**Forward primer (5'>3')**	**Reverse primer (5'>3')**
A0A1Z5RIL8	*SORBI_3005G101700*	Dirigent protein	GCCTTCTTTGACGAGGTCAGG	ACACGTCGATCTTCACAACGG
C5XL59	*SORBI_3003G024700*	Peroxidase	CTCTGTCCGCTGACCTGGAG	TAGAGCTAGGGGACAACGGG
C5YYX3	*SORBI_3009G017800*	Glutathione dehydrogenase (ascorbate)	ATGGAGCTGCTTTTGTGCTTG	AACCTGATGCTCAGGCTATCAC
C5XG44	*SORBI_3003G254300*	Glutaredoxin-dependent peroxiredoxin	GAAACGGTCGTCGTTGAGTC	TCTCAGCATCAACTTTGTGTAAC
C5XQ74	*SORBI_3003G208800*	Aspartic peptidase A1	GAAGGCTGTAAAAGGCGCTG	TTTAACCACGCAGCATGGGC
C5WVD3	*SORBI_3001G193500*	Heat shock 70 kDa protein	AAAGCTGAGGGCATTGATCTG	TTCAACCTTTGCCTTCTCAGC
Reference gene	*Sb03g038910*	Uncharacterized protein	TCCTGAAGCATCTTTCCCTCC	ACAGCCTGATTAGTTGGGGG
Reference gene	*Sb04g003390*	Eukaryotic initiation factor-4A	GATGAGATGCTCTCCCGTGG	TGATCTCTAGGGCCTCTGGG
