# Supplementary material for: Regulation of Proline Accumulation and Protein Secretion in Sorghum under Combined Osmotic and Heat Stress
Source: Plants (Basel). 2024 Jul 6;13(13):1874. doi: 10.3390/plants13131874 (PMC11244414; doi:10.3390/plants13131874)
Supplement: Supplementary file 1 [file plants-13-01874-s001.zip › Table S4.pdf]

Table S4. List of combined osmotic and heat stress-responsive secreted proteins of ICSB338 sorghum cell suspension cultures at 5% significance level.

N[a]	Accession[b]	Protein name[c]	Ratio[d]	SD[e]	p-value[f]	SP[g]	Cellular component[h]	Biological process[i]	Molecular function[j]	Protein family[k]
**CELL WALL MODIFICATION**										
2	C5XYP5	Fibronectin type III-like domain-containing protein OS=*Sorghum bicolor* GN=SORBI_3004G233700	-1.41	0.04	3.31E-03	+	Extracellular region	Xylan catabolic process	Xylan 1,4-beta-xylosidase activity	Beta-D-xylosidase
8	A0A1B6QHZ6	Beta-glucosidase OS=*Sorghum bicolor* GN=SORBI_3001G089000	-1.34	0.10	6.33E-03	-	Extracellular region	Glucan catabolic process	Beta-glucosidase activity	Cellulose degradation glycosyl hydrolase 3
13	A0A1B6QIM7	Uncharacterized protein OS=*Sorghum bicolor* GN=SORBI_3001G123300	-1.49	0.09	1.41E-02	+	None	Carbohydrate metabolic process	Beta-glucosidase activity	Glycoside hydrolase, family 1
16	C5XKE9	Endoglucanase OS=*Sorghum bicolor* GN=SORBI_3003G015700	-1.51	0.12	1.06E-02	+	Extracellular region	Cellulose catabolic process	Cellulase activity	Glycosyl hydrolase, family 9
36	C5Z8N0	FAS1 domain-containing protein OS=*Sorghum bicolor* GN=SORBI_3010G118900	-1.57	0.12	7.97E-03	+	Membrane	Multidimensional cell growth	None	Fasciclin-like arabinogalactan protein
41	C5WXC7	Alpha-galactosidase OS=*Sorghum bicolor* GN=SORBI_3001G208100	-1.83	0.05	1.89E-03	+	Plant-type cell wall	Galactomannan catabolic process	Alpha-galactosidase activity	Glycoside hydrolase, family 27
48	A0A1B6QI05	Beta-glucosidase OS=*Sorghum bicolor* GN=SORBI_3001G089100	1.41	0.19	2.15E-02	+	Extracellular region	Glucan catabolic process	Beta-glucosidase activity	Cellulose degradation glycosyl hydrolase 3
53	A0A1B6Q838	Beta-1,3-glucanase OS=*Sorghum bicolor* GN=SORBI_3003G422200	1.43	0.15	3.96E-02	+	None	Carbohydrate metabolic process	Hydrolase activity, hydrolyzing O-glycosyl compounds	Glycoside hydrolase, family 17
59	C5X4N0	X8 domain-containing protein OS=*Sorghum bicolor* GN=SORBI_3002G255600	1.26	0.15	4.11E-02	+	None	Carbohydrate metabolic process	Glucan endo-1,3-beta-D-glucosidase activity	Glycoside hydrolase, family 17
60	A0A1B6PLT5	DUF642 domain-containing protein OS=*Sorghum bicolor* GN=SORBI_3006G133000	-1.82	0.05	1.25E-02	+	None	None	None	Pectin methylation modulator
67	A0A1W0W3H0	Alpha-galactosidase OS=*Sorghum bicolor* GN=SORBI_3002G123100	-1.69	0.02	3.02E-03	-	Plant-type cell wall	Carbohydrate metabolic process	Alpha-galactosidase activity	Glycoside hydrolase, family 27
88	C5Z8T4	Xyloglucan endotransglucosylase/hydrolase OS=*Sorghum bicolor* GN=SORBI_3010G246600	-1.94	0.07	3.08E-02	+	Extracellular apoplast	Cell wall organisation	Xyloglucan:xyloglucosyl transferase activity	Xyloglucan endotransglucosylase/hydrolas e
98	A0A1B6QE21	UTP--glucose-1-phosphate uridylyltransferase	1.62	0.14	3.56E-04	-	Cytoplasm	UDP-glucose metabolic process	UTP--glucose-1-phosphate	UTP--glucose-1-phosphate uridylyltransferase

		OS=*Sorghum bicolor* GN=SORBI_3002G291200							uridylyltransferase activity	
106	C5XV25	Heparanase-like protein 3 OS=*Sorghum bicolor* GN=SORBI_3004G333600	-1.37	0.06	3.94E-02	+	Plant-type cell wall	None	Beta-glucuronidase activity	Glycoside hydrolase, family 79
155	A0A194YQ33	Beta-fructofuranosidase OS=*Sorghum bicolor* GN=SORBI_3004G166700	1.45	0.18	1.66E-02	+	None	Carbohydrate metabolic process	Hydrolase activity, hydrolyzing O-glycosyl compounds	Glycoside hydrolase, family 32
156	C5XT36	Endoglucanase OS=*Sorghum bicolor* GN=Sb24P17cg_130	-2.11	0.12	7.99E-03	+	None	Cellulose catabolic process	Cellulase activity	Glycosyl hydrolase, family 9
186	C5XRX3	Uncharacterized protein OS=*Sorghum bicolor* GN=SORBI_3004G294500	-2.17	0.14	1.98E-02	+	Extracellular region	Cell wall organisation	None	Expansin/Lol pI
191	A0A1Z5R476	Glucan endo-1,3-beta-D-glucosidase GVI OS=*Sorghum bicolor* GN=SORBI_3009G183400	3.87	0.73	8.10E-04	+	None	Carbohydrate metabolic process	Hydrolase activity, hydrolyzing O-glycosyl compounds	Glycoside hydrolase, family 17
234	C5XIT5	Pectinesterase OS=*Sorghum bicolor* GN=SORBI_3003G148300	-1.56	0.07	7.40E-03	+	Extracellular region	Cell wall modification	Pectinesterase activity	Pectinesterase family
299	C5XU04	Pectinesterase inhibitor domain-containing protein OS=*Sorghum bicolor* GN=SORBI_3004G167700	3.30	1.49	3.77E-02	+	Apoplast	None	Pectinesterase inhibitor activity	Pla a 1-like
305	C5YSN7	Beta-galactosidase OS=*Sorghum bicolor* GN=SORBI_3008G052200	1.25	0.11	2.51E-02	+	Apoplast	Carbohydrate metabolic process	Beta-galactosidase activity	Glycoside hydrolase, family 35
**DEFENCE/OXIDATIVE STRESS RESPONSE**										
3	A0A1W0W7T8	Peroxidase OS=*Sorghum bicolor* GN=SORBI_3002G416600	-1.63	0.09	1.37E-02	+	Extracellular region	Responsive to oxidative stress	Peroxidase activity	Plant peroxidase
7	C5Y360	Peroxidase OS=*Sorghum bicolor* GN=SORBI_3005G011300	-1.68	0.10	1.91E-02	+	Extracellular region	Responsive to oxidative stress	Peroxidase activity	Plant peroxidase
12	C5Z475	Peroxidase OS=*Sorghum bicolor* GN=SORBI_3010G162000	-1.74	0.07	2.95E-02	+	Extracellular region	Responsive to oxidative stress	Peroxidase activity	Plant peroxidase
21	C5WYQ4	Peroxidase OS=*Sorghum bicolor* GN=SORBI_3001G360400	-1.81	0.08	1.38E-03	+	Extracellular region	Responsive to oxidative stress	Peroxidase activity	Plant peroxidase
29	C5X5K6	Peroxidase OS=*Sorghum bicolor* GN=SORBI_3002G416700	-1.49	0.07	1.93E-05	+	Extracellular region	Responsive to oxidative stress	Peroxidase activity	Plant peroxidase
30	C5XB39	GH18 domain-containing protein OS=*Sorghum bicolor*	2.69	0.54	1.95E-03	+	Extracellular region	Carbohydrate metabolic process	Chitinase activity	Glycoside hydrolase 18 family chitinases

		GN=SORBI_3002G055700								
33	C5YBE9	Chitinase<br>OS=*Sorghum bicolor*<br>GN=SORBI_3006G132400	-1.37	0.12	4.94E-02	+	None	Chitin catabolic process	Chitinase activity	Glycoside hydrolase, family 19
38	C5XJT8	Protein disulfide-isomerase<br>OS=*Sorghum bicolor*<br>GN=SORBI_3003G156400	2.93	0.73	3.88E-03	+	Endoplasmic reticulum	Protein folding	Protein disulfide isomerase activity	Protein disulfide isomerase
39	A0A1B6PQR2	Protein disulfide-isomerase<br>OS=*Sorghum bicolor*<br>GN=SORBI_3005G074400	2.08	0.44	6.89E-03	+	Endoplasmic reticulum	Protein folding	Protein disulfide isomerase activity	Protein disulfide isomerase
50	C5XB38	GH18 domain-containing protein<br>OS=*Sorghum bicolor*<br>GN=SORBI_3002G055600	1.41	0.14	5.14E-03	+	Extracellular region	Carbohydrate metabolic process	Chitinase activity	Glycoside hydrolase 18 family chitinases
54	A0A1B6QFT1	Peroxidase<br>OS=*Sorghum bicolor*<br>GN=SORBI_3002G392000	-2.24	0.08	2.57E-04	+	Extracellular region	Responsive to oxidative stress	Peroxidase activity	Plant peroxidase
57	C5XL59	Peroxidase<br>OS=*Sorghum bicolor*<br>GN=SORBI_3003G024700	-2.93	0.06	1.62E-03	-	Extracellular region	Responsive to oxidative stress	Peroxidase activity	Plant peroxidase
71	A0A1W0W7I8	Plant heme peroxidase family profile domain-containing protein<br>OS=*Sorghum bicolor*<br>GN=SORBI_3002G391900	-1.62	0.12	3.36E-02	-	Plant-type cell wall	Responsive to oxidative stress	Peroxidase activity	Plant peroxidase
76	C5XIY1	Peroxidase<br>OS=*Sorghum bicolor*<br>GN=SORBI_3003G152100	-1.42	0.05	1.75E-02	+	Extracellular region	Responsive to oxidative stress	Peroxidase activity	Plant peroxidase
84	C5X3C1	Peroxidase<br>OS=*Sorghum bicolor*<br>GN=SORBI_3002G391300	-2.25	0.08	9.64E-03	+	Extracellular region	Responsive to oxidative stress	Peroxidase activity	Plant peroxidase
90	C5YBE8	Chitinase<br>OS=*Sorghum bicolor*<br>GN=SORBI_3006G132300	1.59	0.21	1.95E-02	+	None	Chitin catabolic process	Chitinase activity	Glycoside hydrolase, family 19
91	C5Z469	Peroxidase<br>OS=*Sorghum bicolor*<br>GN=SORBI_3010G161600	-1.82	0.13	1.90E-02	+	Extracellular region	Responsive to oxidative stress	Peroxidase activity	Plant peroxidase
102	C5X040	Peroxidase<br>OS=*Sorghum bicolor*<br>GN=SORBI_3001G080300	1.40	0.23	3.32E-02	+	Extracellular region	Responsive to oxidative stress	Peroxidase activity	Plant peroxidase
113	C5X3C6	Peroxidase<br>OS=*Sorghum bicolor*<br>GN=SORBI_3002G391800	-2.09	0.08	1.45E-03	+	Extracellular region	Responsive to oxidative stress	Peroxidase activity	Plant peroxidase
130	C5X0X1	Peroxidase<br>OS=*Sorghum bicolor*<br>GN=SORBI_3001G528100	1.29	0.09	1.17E-02	+	Extracellular region	Responsive to oxidative stress	Peroxidase activity	Plant peroxidase

135	C5XN52	Thaumatin-like protein OS=*Sorghum bicolor* GN=SORBI_3003G331700	2.00	0.43	7.06E-03	+	Extracellular region	Defense response	None	Thaumatin family
138	A0A1B6QGB6	Peroxidase OS=*Sorghum bicolor* GN=SORBI_3002G416800	-2.07	0.07	2.27E-02	+	Extracellular region	Responsive to oxidative stress	Peroxidase activity	Plant peroxidase
144	C5YQ75	Peroxidase OS=*Sorghum bicolor* GN=SORBI_3008G010500	-1.57	0.06	1.14E-03	+	Extracellular region	Responsive to oxidative stress	Peroxidase activity	Plant peroxidase
161	A0A1B6Q537	Chitinase OS=*Sorghum bicolor* GN=SORBI_3003G244600	2.05	0.49	1.02E-02	-	Extracellular region	Carbohydrate metabolic process	Chitinase activity	Glycoside hydrolase 18 family chitinases
173	C5XYY5	Peroxidase OS=*Sorghum bicolor* GN=SORBI_3004G105100	-1.87	0.23	3.80E-02	+	Extracellular region	Responsive to oxidative stress	Peroxidase activity	Plant peroxidase
184	C6JSB7	Peroxidase OS=*Sorghum bicolor* GN=Sb0246s002010	-1.82	0.05	2.15E-02	+	Extracellular region	Responsive to oxidative stress	Peroxidase activity	Plant peroxidase
192	C5YHR8	Peroxidase OS=*Sorghum bicolor* GN=SORBI_3007G192300	-1.40	0.05	3.56E-03	+	Extracellular region	Responsive to oxidative stress	Peroxidase activity	Plant peroxidase
212	A0A194YM75	Uncharacterized protein OS=*Sorghum bicolor* GN=SORBI_3004G011700	1.61	0.21	1.46E-02	+	Endoplasmic reticulum	Protein folding	Heat shock protein binding	Heat shock protein 70 family
215	C5YZJ2	Peroxidase OS=*Sorghum bicolor* GN=SORBI_3009G033400	-1.61	0.18	3.93E-02	+	Extracellular region	Responsive to oxidative stress	Peroxidase activity	Plant peroxidase
237	C5XHX2	Germin-like protein OS=*Sorghum bicolor* GN=SORBI_3003G427700	2.09	0.47	1.16E-02	+	Apoplast	Plasmodesmata-mediated intercellular transport	Manganese ion binding	Germin
241	C5WVD3	Heat shock 70 kDa protein, mitochondrial OS=*Sorghum bicolor* GN=SORBI_3001G193500	3.99	1.37	9.44E-03	-	Mitochondrion	Protein folding	Heat shock protein binding	Heat shock protein 70 family
246	C5YVR0	Superoxide dismutase OS=*Sorghum bicolor* GN=SORBI_3009G093200	2.78	0.07	1.27E-06	-	Mitochondrion	Removal of superoxide radicals	Superoxide dismutase activity	Manganese/iron superoxide dismutase
250	C5YYX3	Glutathione dehydrogenase (ascorbate) OS=*Sorghum bicolor* GN=SORBI_3009G017800	2.58	0.23	1.25E-04	-	None	Cellular oxidant detoxification	Glutathione dehydrogenase (ascorbate) activity	Dehydroascorbate reductases DHAR1/2/3/4
260	C5XG44	Glutaredoxin-dependent peroxiredoxin OS=*Sorghum bicolor* GN=SORBI_3003G254300	1.78	0.39	2.55E-02	-	Cytoplasm	Cellular oxidant detoxification	Thioredoxin peroxidase activity	Peroxiredoxin-5-like
264	C5XCE2	Zeamatin-like protein OS=*Sorghum bicolor* GN=SORBI_3002G351400	1.78	0.35	1.07E-02	+	Extracellular region	Defense response	None	Thaumatin family

282	A0A1B6QA33	Calreticulin OS=*Sorghum bicolor* GN=SORBI_3002G090500	1.97	0.20	2.23E-04	+	Endoplasmic reticulum membrane	Protein folding	Unfolded protein binding	Calreticulin
289	A0A1B6QG28	Superoxide dismutase [Cu-Zn] OS=*Sorghum bicolor* GN=SORBI_3002G407900	3.26	0.75	2.39E-03	-	None	Removal of superoxide radicals	Superoxide dismutase activity	Superoxide dismutase [Cu-Zn]/ superoxide dismutase copper chaperone
298	C5YBF0	Chitinase OS=*Sorghum bicolor* GN=SORBI_3006G132500	1.90	0.40	1.44E-02	+	None	Chitin catabolic process	Chitinase activity	Glycoside hydrolase, family 19
331	C5WRK4	Small nuclear ribonucleoprotein Sm D3 OS=*Sorghum bicolor* GN=SORBI_3001G440900	-2.49	0.20	3.28E-02	-	Cytosol	Spliceosomal snRNP assembly	RNA binding	Small nuclear ribonucleoprotein Sm D3
359	A0A1Z5RIL8	Dirigent protein OS=*Sorghum bicolor* GN=SORBI_3005G101700	1.94	0.28	3.53E-03	+	Apoplast	Phenylpropanoid biosynthetic process	None	Dirigent protein
393	A0A1B6Q818	Glutathione transferase OS=*Sorghum bicolor* GN=SORBI_3003G416300	1.89	0.33	4.12E-03	-	Cytoplasm	Glutathione metabolic process	Glutathione transferase activity	Glutathione-S-transferase
414	C5YXM1	Dienelactone hydrolase domain-containing protein OS=*Sorghum bicolor* GN=SORBI_3009G129800	1.72	0.30	8.64E-03	-	None	None	Hydrolase activity	Dienelactone hydrolase family
420	C5Z0N8	Peroxidase OS=*Sorghum bicolor* GN=SORBI_3009G055100	-1.74	0.20	3.11E-02	+	Extracellular region	Responsive to oxidative stress	Peroxidase activity	Plant peroxidase
476	A0A1B6QN96	Superoxide dismutase [Cu-Zn] OS=*Sorghum bicolor* GN=SORBI_3001G371900	2.70	0.85	1.46E-02	-	Extracellular space	Removal of superoxide radicals	Superoxide dismutase activity	Superoxide dismutase (Cu/Zn)/ superoxide dismutase copper chaperone
651	C5YXM3	Dienelactone hydrolase domain-containing protein OS=*Sorghum bicolor* GN=SORBI_3009G130000	1.73	0.20	1.56E-02	-	None	None	Hydrolase activity	Dienelactone hydrolase family
656	C5Z4L3	Thaumatin-like protein 1 OS=*Sorghum bicolor* GN=SORBI_3010G166700	2.80	1.02	2.93E-02	+	Extracellular region	Defense response	None	Thaumatin family
740	C5YQ75	Peroxidase OS=*Sorghum bicolor* GN=SORBI_3008G010500	-1.57	0.05 6	1.14E-03	+	Extracellular region	Responsive to oxidative stress	Peroxidase activity	Plant peroxidase
762	C5Z0K4	Protein disulfide-isomerase OS=*Sorghum bicolor* GN=SORBI_3009G051600	2.22	0.83	4.72E-02	+	Endoplasmic reticulum	Protein folding	Protein disulfide isomerase activity	Protein disulfide isomerase
**PROTEOLYSIS**										
1	C5Y8G7	Peptidase A1 domain-containing protein OS=*Sorghum bicolor* GN=SORBI_3005G224500	-1.35	0.04	3.47E-02	+	None	Proteolysis	Aspartic-type endopeptidase activity	Aspartic peptidase A1 family

15	C5Z6U2	Ubiquitin-like domain-containing protein OS=*Sorghum bicolor* GN=SORBI_3010G210000	1.97	0.30	3.34E-03	-	Cytoplasm	Protein ubiquitination	Ubiquitin protein ligase binding	Ubiquitin and ubiquitin-like
22	C5Y675	Peptidase A1 domain-containing protein OS=*Sorghum bicolor* GN=SORBI_3005G064200	-1.59	0.03	2.58E-03	+	None	Proteolysis	Aspartic-type endopeptidase activity	Aspartic peptidase A1 family
40	C5XQ74	Peptidase A1 domain-containing protein OS=*Sorghum bicolor* GN=SORBI_3003G208800	-1.66	0.08	2.43E-03	+	None	Proteolysis	Aspartic-type endopeptidase activity	Aspartic peptidase A1 family
72	C5X3T4	Peptidase A1 domain-containing protein OS=*Sorghum bicolor* GN=SORBI_3002G243000	-1.84	0.07	3.09E-05	+	None	Proteolysis	Aspartic-type endopeptidase activity	Aspartic peptidase A1 family
108	A0A1B6Q6M7	Cysteine proteinase inhibitor OS=*Sorghum bicolor* GN=SORBI_3003G327700	1.71	0.35	2.39E-02	+	None	None	Cysteine-type endopeptidase inhibitor activity	Cystatin
119	C5XQP2	Peptidase A1 domain-containing protein OS=*Sorghum bicolor* GN=SORBI_3003G078400	-1.39	0.02	8.17E-03	+	None	Proteolysis	Aspartic-type endopeptidase activity	Aspartic peptidase A1 family
162	A0A1B6PEH1	Peptidase A1 domain-containing protein OS=*Sorghum bicolor* GN=SORBI_3008G184400	1.41	0.12	1.17E-02	+	None	Proteolysis	Aspartic-type endopeptidase activity	Aspartic peptidase A1 family
232	C5XG67	Cystatin domain-containing protein OS=*Sorghum bicolor* GN=SORBI_3003G400400	2.76	0.70	6.17E-03	+	None	None	Cysteine-type endopeptidase inhibitor activity	Cystatin
270	C5XG88	Small ubiquitin-related modifier OS=*Sorghum bicolor* GN=SORBI_3003G402600	2.68	0.83	1.64E-02	-	Nucleus	Protein sumoylation	Ubiquitin-like protein ligase binding	Ubiquitin-like domain superfamily
321	C5YCI1	Alpha-amylase/subtilisin inhibitor OS=*Sorghum bicolor* GN=SORBI_3006G154300	1.42	0.14	3.57E-02	+	None	None	Serine-type endopeptidase inhibitor activity	Proteinase inhibitor I3, Kunitz legume
363	A0A1B6P6G7	Aspartic proteinase OS=*Sorghum bicolor* GN=SORBI_3009G034700	-2.00	0.11	7.05E-03	+	None	Proteolysis	Aspartic-type endopeptidase activity	Aspartic peptidase A1 family
626	A0A1B6PA25	Peptidase A1 domain-containing protein OS=*Sorghum bicolor* GN=SORBI_3009G234800	-1.33	0.11	3.50E-02	-	Lysosome	Proteolysis	Aspartic-type endopeptidase activity	Aspartic peptidase A1 family
**METABOLISM**										

23	C5XX52	Glyceraldehyde-3-phosphate dehydrogenase OS=*Sorghum bicolor* GN=SORBI_3004G205100	-1.47	0.11	3.62E-03	-	Cytosol	Glycolytic process	Glyceraldehyde-3-phosphate dehydrogenase (NAD+) (phosphorylating) activity	Glyceraldehyde-3-phosphate dehydrogenase, type I
28	C5Z240	Monocopper oxidase-like protein SKU5 OS=*Sorghum bicolor* GN=SORBI_3010G003100	-1.34	0.12	4.19E-02	+	None	None	Oxidoreductase activity	Multicopper oxidase
55	C5X9Y2	L-ascorbate oxidase OS=*Sorghum bicolor* GN=SORBI_3002G314800	-1.34	0.10	3.61E-02	+	None	None	Oxidoreductase activity	Multicopper oxidase
61	A0A194YMV2	Phosphoglycerate kinase OS=*Sorghum bicolor* GN=SORBI_3004G055200	2.12	0.30	9.56E-04	-	Cytosol	Glycolytic process	Phosphoglycerate kinase activity	Phosphoglycerate kinase
69	C5Y9T3	Aldose 1-epimerase OS=*Sorghum bicolor* GN=SORBI_3006G105200	-1.36	0.06	8.43E-03	+	None	Hexose metabolic process	Aldose 1-epimerase activity	Aldose 1-epimerase
92	A0A1Z5RC44	Glycerophosphodiester phosphodiesterase OS=*Sorghum bicolor* GN=SORBI_3007G213300	-1.37	0.15	4.03E-02	-	Membrane	Lipid metabolic process	Glycerophosphodiester phosphodiesterase activity	Glycerophosphoryl diester phosphodiesterase
137	A0A1W0VY92	GDSL esterase/lipase OS=*Sorghum bicolor* GN=SORBI_3003G205900	-1.99	0.04	7.30E-03	+	None	None	Hydrolase activity, acting on ester bonds	GDSL lipase/esterase -like, plant
151	A0A1B6PEZ5	Carbohydrate kinase PfkB domain-containing protein OS=*Sorghum bicolor* GN=SORBI_3007G014700	-1.43	0.20	4.99E-02	-	Cytosol	Fructose metabolic process	Fructokinase	PfkB carbohydrate kinase
171	C5WYF2	Malate dehydrogenase OS=*Sorghum bicolor* GN=SORBI_3001G219300	1.73	0.37	1.63E-02	-	Cytosol	Malate metabolic process	L-malate dehydrogenase activity	Malate dehydrogenase NAD-dependent, cytosolic
175	C5X780	Phytocyanin domain-containing protein OS=*Sorghum bicolor* GN=SORBI_3002G007200	-1.61	0.10	2.64E-02	+	None	None	Electron transfer activity	Phytocyanin-like
231	A0A194YIA9	Esterase OS=*Sorghum bicolor* GN=SORBI_3010G044500	-2.40	0.08	3.03E-02	+	None	None	Hydrolase activity, acting on ester bonds	GDSL lipase/esterase-like, plant
257	C5Z861	Phytocyanin domain-containing protein OS=*Sorghum bicolor* GN=SORBI_3010G231900	-1.48	0.06	4.79E-03	+	Membrane	None	Electron transfer activity	Phytocyanin-like
273	A0A1B6Q670	Aspartate aminotransferase OS=*Sorghum bicolor* GN=SORBI_3003G303300	1.55	0.29	2.05E-02	-	Cytosol	Amino acid biosynthetic process	Transaminase activity	Aspartate/other aminotransferase
308	C5Z4E5	Esterase OS=*Sorghum bicolor* GN=SORBI_3010G044900	-2.77	0.11	3.64E-04	+	None	None	Hydrolase activity, acting on ester bonds	GDSL lipase/esterase-like, plant

349	A0A1Z5SAM0	Allene oxide synthase OS=*Sorghum bicolor* GN=SORBI_3001G449700	-4.43	0.29	4.32E-02	-	None	Sterol metabolic process	Monooxygenase activity	Cytochrome P450
385	C5WYI7	Cyanate hydratase OS=*Sorghum bicolor* GN=CYN	2.00	0.56	3.44E-02	-	None	Cyanate catabolic process	Cyanate hydratase activity	Cyanate hydratase
418	C5YW21	Malate dehydrogenase OS=*Sorghum bicolor* GN=SORBI_3009G240700	2.02	0.54	3.75E-02	-	Cytoplasm	Malate metabolic process	L-malate dehydrogenase activity	Malate dehydrogenase, type 1
484	A0A1Z5RI82	Enoyl reductase (ER) domain-containing protein OS=*Sorghum bicolor* GN=SORBI_3005G082700	2.52	0.44	1.02E-02	-	None	None	Oxidoreductase activity	Medium-chain dehydrogenase/reductase
469	A0A194YGY2	Phosphopyruvate hydratase OS=*Sorghum bicolor* GN=SORBI_3010G027000	1.39	0.15	3.25E-02	-	Phosphopyruvate hydratase complex	Glycolytic process	Phosphopyruvate hydratase activity	Enolase
219	C5XIY9	Dihydrolipoyl dehydrogenase OS=*Sorghum bicolor* GN=SORBI_3003G152900	2.19	0.51	1.02E-02	-	Mitochondrion	None	Dihydrolipoyl dehydrogenase activity	Dihydrolipoamide dehydrogenase
221	C5Z0L0	Dihydrolipoyl dehydrogenase OS=*Sorghum bicolor* GN=SORBI_3009G052200	1.94	0.64	4.91E-02	-	Mitochondrion	None	Dihydrolipoyl dehydrogenase activity	Dihydrolipoamide dehydrogenase
**SIGNAL TRANSDUCTION**										
107	C5WRH5	Nucleoside diphosphate kinase OS=*Sorghum bicolor* GN=SORBI_3001G295200	1.53	0.26	3.40E-02	-	None	Phosphorylation	Nucleoside diphosphate kinase activity	Nucleoside diphosphate kinase
332	C5WPY7	Protein kinase domain-containing protein OS=*Sorghum bicolor* GN=SORBI_3001G277500	-1.85	0.10	2.13E-03	+	Plasma membrane	Phosphorylation	Protein serine/threonine kinase activity	Protein tyrosine and serine/threonine kinase
357	C5XQS6	EF-hand domain-containing protein OS=*Sorghum bicolor* GN=SORBI_3003G082600	3.67	0.13	6.94E-03	-	None	None	Calcium ion binding	None
**UNCLASSIFIED**										
9	A0A1B6PKE9	FAD-binding PCMH-type domain-containing protein OS=*Sorghum bicolor* GN=SORBI_3006G056300	-1.65	0.12	1.85E-02	+	None	None	Oxidoreductase activity	Oxygen-dependent FAD-linked oxidoreductase
25	C5Z484	FAD-binding PCMH-type domain-containing protein OS=*Sorghum bicolor* GN=SORBI_3010G163000	1.24	0.06	1.22E-02	+	None	None	Oxidoreductase activity	Oxygen-dependent FAD-linked oxidoreductase
26	C5XBP7	Leucine-rich repeat-containing N-terminal plant-type domain-containing protein	-3.70	0.05	3.06E-05	+	None	Specification of floral organ number	Enzyme inhibitor activity	Polygalacturonase-inhibiting protein

		OS=*Sorghum bicolor* GN=SORBI_3002G343600								
43	A0A1Z5R915	Purple acid phosphatase OS=*Sorghum bicolor* GN=SORBI_3007G091100	-2.04	0.04	6.53E-03	-	None	None	Acid phosphatase activity	Purple acid phosphatase
126	C5Z6Y0	Uncharacterized protein OS=*Sorghum bicolor* GN=SORBI_3010G088700	-1.66	0.06	1.11E-03	+	Apoplast	None	None	Protein exordium-like
154	C5XCI9	Glucose/Sorbosone dehydrogenase domain-containing protein OS=*Sorghum bicolor* GN=SORBI_3002G068000	1.39	0.19	1.90E-02	+	Membrane	None	None	Glucose/Sorbosone dehydrogenase
158	C5X4M5	DOMON domain-containing protein OS=*Sorghum bicolor* GN=SORBI_3002G255000	-2.45	0.11	1.46E-03	+	Membrane	None	None	Protein of unknown function (DUF568)
255	C5XYB7	Uncharacterized protein OS=*Sorghum bicolor* GN=SORBI_3004G229500	-3.59	0.08	1.67E-02	+	Apoplast	None	None	Protein exordium-like
295	C5XL53	Peptide-N4-(N-acetyl-beta-glucosaminyl)asparagine amidase A OS=*Sorghum bicolor* GN=SORBI_3003G024000	1.54	0.26	1.41E-02	+	None	None	None	Peptide-N4-(N-acetyl-beta-glucosaminyl) asparagine amidase A
342	C5XYB4	Phi-1-like phosphate-induced protein OS=*Sorghum bicolor* GN=SORBI_3004G229300	-1.55	0.11	2.36E-02	+	Apoplast	None	None	Protein exordium-like
211	C5Y2R8	Leucine-rich repeat-containing N-terminal plant type domain-containing protein OS=*Sorghum bicolor* GN=SORBI_3005G126200	3.81	0.31	8.46E-06	+	None	None	Protein binding	Leucine-rich repeat-containing N-terminal plant type

[a]Protein number (N) assigned in ProteinPilot.
[b]Protein accession numbers obtained from the UniProt database searches against sequences of *Sorghum bicolor* only.
[c]Protein name retrieved from Uniprot database on 31 March 2024.
[d]Ratio represents the average fold-change (n = 4) in response to combined osmotic and heat stress relative to the control. A positive value indicates up-regulation, while a negative value indicates down-regulation.
[e]Standard deviation of the fold-changes (n = 4).
[f]Probability value obtained from a Student's t-test comparing the fold changes between the combined osmotic and heat stress treatment and the control (n = 4).
[g]Signal peptide (SP) prediction results for each protein as determined by the SignalP 6.0 server (https://services.healthtech.dtu.dk/services/SignalP-6.0/). + indicated presence of a signal peptide, while – indicates absence of a signal peptide.
[h-j]Gene Ontology terms for each protein as collated from the UniProt database.
[k]Family name as predicted using the InterPro (http://www.ebi.ac.uk/interpro/) or Pfam (http://pfam.xfam.org) databases.
