# Supplementary figures and images for: Regulation of Proline Accumulation and Protein Secretion in Sorghum under Combined Osmotic and Heat Stress
Source: Plants (Basel). 2024 Jul 6;13(13):1874. doi: 10.3390/plants13131874 (PMC11244414; doi:10.3390/plants13131874)

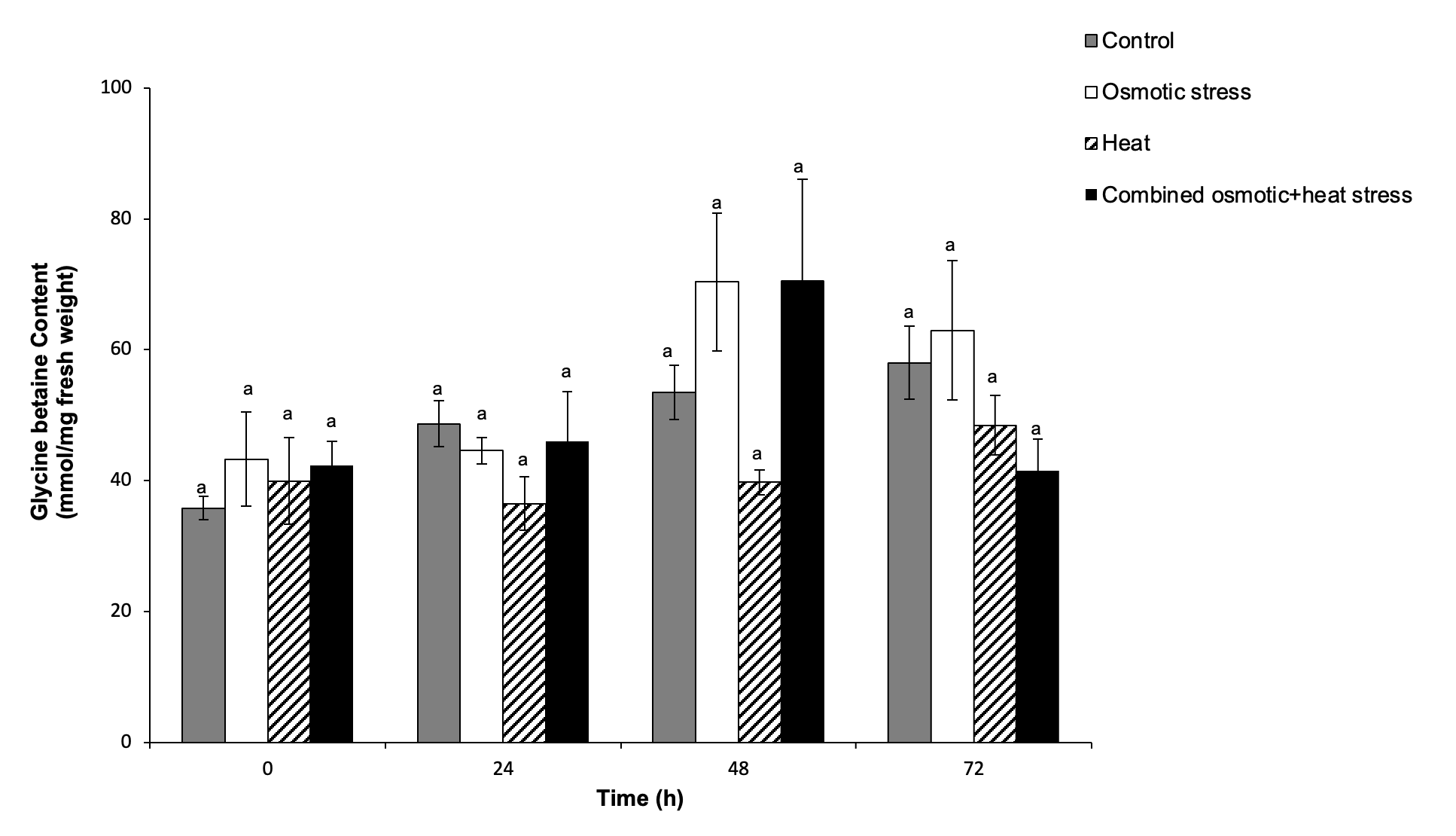

Supplement: Supplementary file 1 [file plants-13-01874-s001.zip › Figure S1.png]
